# Supplementary material for: Ectopic expression of HIV-1 Tat modifies gene expression in cultured B cells: implications for the development of B-cell lymphomas in HIV-1-infected patients
Source: PeerJ. 2022 Oct 18;10:e13986. doi: 10.7717/peerj.13986 (PMC9586123; doi:10.7717/peerj.13986)
Supplement: Supplemental Information 1 [file peerj-10-13986-s001.pdf]

Supplementary Figures

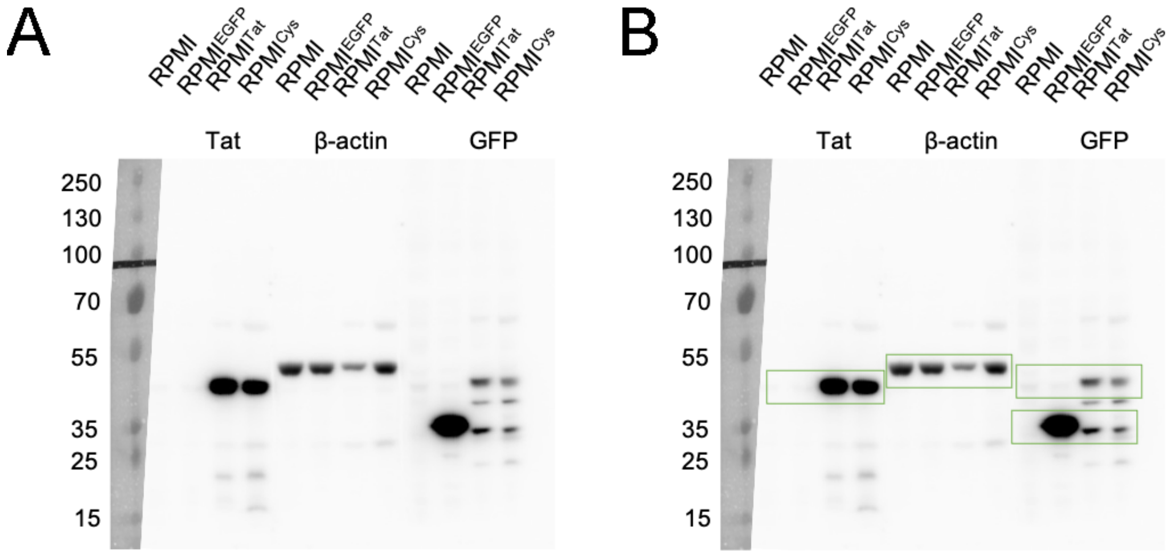

Figure S1. Nonprocessed images of the western blot experiment presented in Fig. 1C. (A) Nonprocessed image. (B) Image with the cropped regions of interest, which are presented in Fig. 1C.

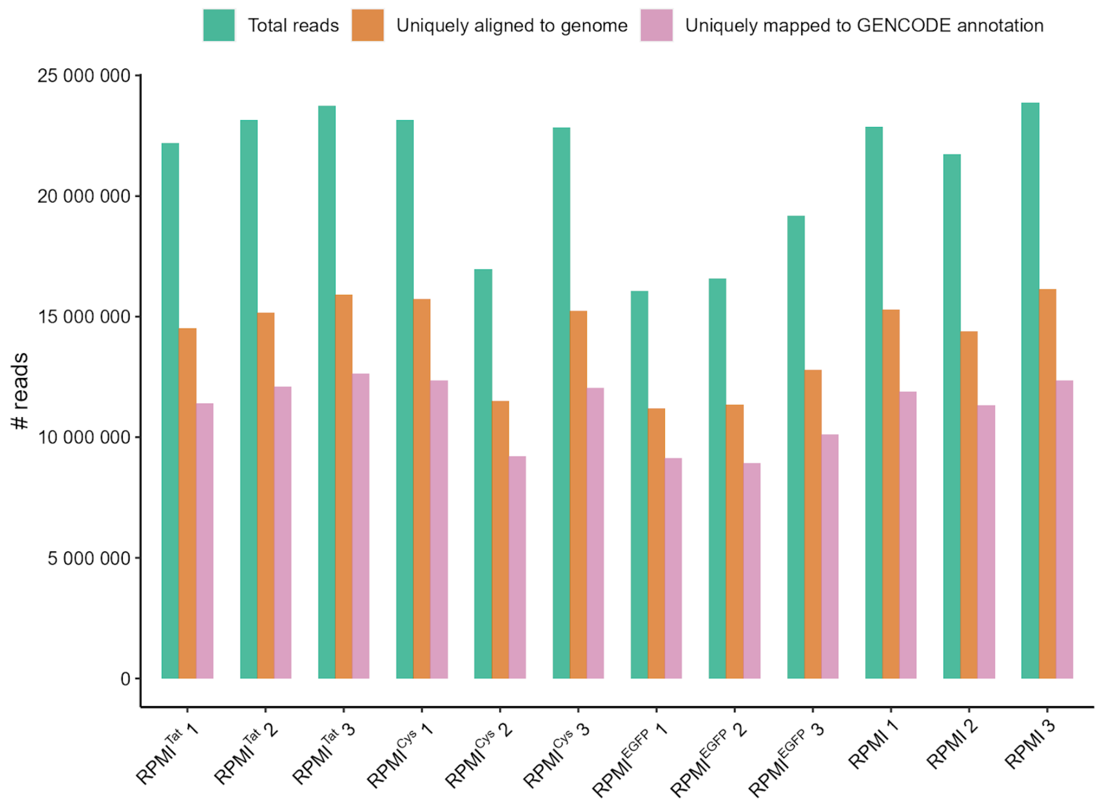

Figure S2. The number of reads at different stages of RNA-seq analysis: total reads obtained from RNA sequencing, reads that were uniquely aligned to the reference human genome GRCh38.p10, and reads that were unambiguously mapped to the GENCODE v26 gene annotation.

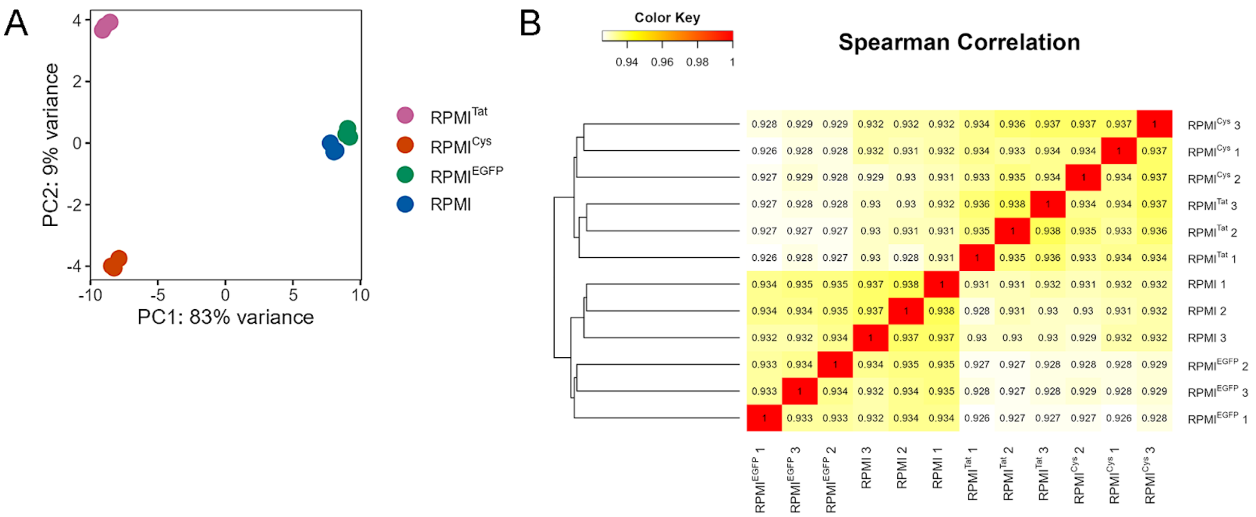

Figure S3. Quality control of the RNA-seq replicates. (A) PCA performed on regularized log-transformed filtered count data. (B) Heatmap of Spearman correlation coefficients between DESeq2-normalized gene expression profiles.

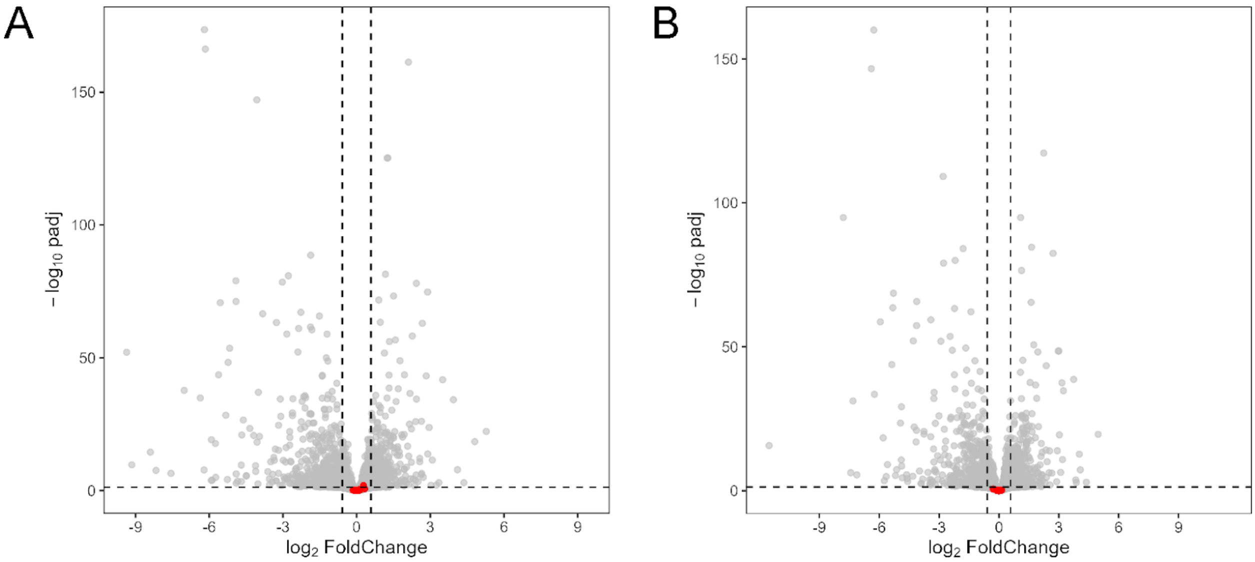

Figure S4. Tat protein had no impact on the expression of EBV genes in RPMI^{Tat} cells. The volcano plot shows the logarithm of the fold change in gene expression (along the X-axis) and the significance of the gene expression change (along the Y-axis). EBV genes are highlighted in red, and human genes are shown as gray dots. (A) RPMI^{Tat} cells. (B) RPMI^{Cys} cells.

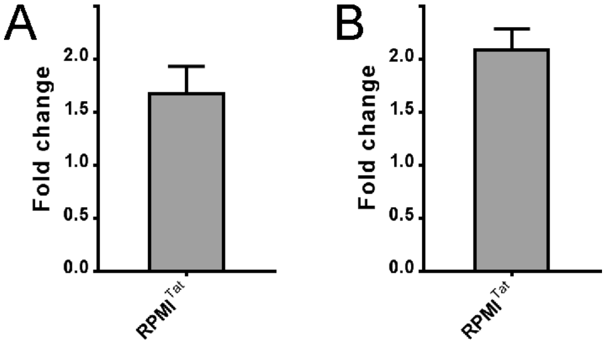

Figure S5. Validation of the RNA-seq dataset using qRT-PCR (mean ± SEM; n = 3). (A) MALAT1 gene. (B) NEAT1 gene.
