# Supplementary material for: Ectopic expression of HIV-1 Tat modifies gene expression in cultured B cells: implications for the development of B-cell lymphomas in HIV-1-infected patients
Source: PeerJ. 2022 Oct 18;10:e13986. doi: 10.7717/peerj.13986 (PMC9586123; doi:10.7717/peerj.13986)
Supplement: Supplemental Information 3 [file peerj-10-13986-s003.pdf]

Table S2. Primers used in this study.

NEAT 1_2	5’-GCTTATCCCACAGCACACAC-3’<br>5’-CTAAGTCCCAACCTGCCGAT-3’
MALAT1	5’-GACGGAGGTTGAGATGAAGC-3’<br>5’-ATTCGGGGCTCTGTAGTCCT-3’
UBC	5’-GTCGCAGCCGGGATTTGGGT-3’<br>5’-GTCAGACAGGGTGCGCCCAT-3’
YWHAZ	5’-ACTTTTGGTACATTGTGGCTTCAA-3’<br>5’-CCGCCAGGACAAACCAGTAT-3’
HPRT1	5’-TGTAATGACCAGTCAACAGGGGACA-3’<br>5’-TCCAACACTTCGTGGGGTCCT-3’
IL6R	5’-CTCCTGCCAGTTAGCAGTCC-3’<br>5’-TCTTGCCAGGTGACACTGAG-3’
IL2RB	5’-GCCCCCATCTCCCTCCAAGT-3’<br>5’-AGGGGAAGGGCGAAGAGAGC-3’
CCR4	5’-TGTTCACTGCTGCCTTAATCCCATC-3’<br>5’-TGGACTGCGTGTAAGATGAGCTGG-3’
IL1R	5’-CCTGCTATGATTTTCTCCCAATAAA-3’<br>5’-<br>AACACAAAAATATCACAGTCAGAGGTAGAC-3’
IL21R	5’-GTGTGGATTCTCACCCCAGGC-3’<br>5’-TGCCTCATCTTCTGTTTCGGCT-3’
IL27R	5’-GTCTGCATGAATGTGAGTGGC-3’<br>5’-AACAGGAACAAGCCCCACAA-3’
IL17R	5’-GAGAGCCGACCGTTCAATGT-3’<br>5’-CTTCAACAAGCGGATGCTGG-3’
TLR8	5’-TGGGAAAGGAGACTAAAAAGGAAA-3’<br>5’-TCTGGTGCTGTACATTGGGG-3’

NLRP4	5'-CCTGGTATACCTGATGTTGGCT-3'<br>5'-TCTCCGATTTCATTGCACCCA-3'
CCL3L1	5'-AGGTCCTCTCTGCACCACTT-3'<br>5'-CTCTCTTGGTTAGGAAGATGACACT-3'
TLR7	5'-TGCCATCAAGAAAGTTGATGCTAT-3'<br>5'-CAGAGTGACATCACAGGGCA-3'
STAT1	5'-GAACGGAGGCGAACCTGACT-3'<br>5'-GCAAGGCTGGCTTGAGGTT-3'
