# Supplementary material for: Ectopic expression of HIV-1 Tat modifies gene expression in cultured B cells: implications for the development of B-cell lymphomas in HIV-1-infected patients
Source: PeerJ. 2022 Oct 18;10:e13986. doi: 10.7717/peerj.13986 (PMC9586123; doi:10.7717/peerj.13986)
Supplement: Supplemental Information 4 [file peerj-10-13986-s004.pdf]

Table S3

Differentially expressed zinc finger proteins (extracted from UniprotKB database).

Uniprot ID	Protein Names	Gene Names	(Suggested) function	References
P17017	**Zinc finger protein 14**	***ZNF14***	May be involved in transcriptional regulation.	(Thiesen, 1990; Grimwood et al., 2004; Gerhard et al., 2004)
P17031	**Zinc finger protein 26**	***ZNF26***	May be involved in transcriptional regulation.	(Thiesen, 1990; Ota et al., 2004; Gerhard et al., 2004; Scherer et al., 2006)
P17039	**Zinc finger protein 30**	***ZNF30***	May be involved in transcriptional regulation.	(Thiesen, 1990; Ota et al., 2004; Grimwood et al., 2004; Gerhard et al., 2004; Bechtel et al., 2007)
P51523	**Zinc finger protein 84**	***ZNF84***	May be involved in transcriptional regulation.	(Bellefroid et al., 1989; Rosati et al., 1991; Ota et al., 2004; Gerhard et al., 2004; Rigbolt et al., 2011; Zhou et al., 2013; Hendriks et al., 2017)
P52738	**Zinc finger protein 140**	***ZNF140***	May be involved in transcriptional regulation as a repressor.	(Tommerup & Vissing, 1995; Vissing et al., 1995; Ota et al., 2004; Gerhard et al., 2004; Scherer et al., 2006)
Q12901	**Zinc finger protein 155**	***ZNF155***	May be involved in transcriptional regulation.	(Tommerup & Vissing, 1995; Shannon et al., 2003; Ota et al., 2004; Grimwood et al., 2004; Gerhard et al., 2004; Sjöblom et al., 2006)
Q2M3W8	**Zinc finger protein 181**	***ZNF181***	May be involved in transcriptional regulation.	(Grimwood et al., 2004; Gerhard et al., 2004; Hendriks et al., 2014, 2017)

Q75346	**Zinc finger protein 253**	***ZNF253***	May function as a transcription factor. Seems to have a transcriptional repression activity.	(Han et al., 1999; Ota et al., 2004; Grimwood et al., 2004; Gerhard et al., 2004)
Q14587	**Zinc finger protein 268**	***ZNF268***	Isoform 1<br>Acts as a transcriptional repressor. Inhibits erythroid differentiation and tumor cell proliferation. Plays a role during ovarian cancer development and progression.<br>Isoform 2<br>Contributes to cervical carcinogenesis in part through the TNF-alpha-induced NF-kappa-B signaling pathway by interacting with the I-kappa-B-kinase (IKK) core complex.	(Abrink, Aveskogh & Hellman, 1995; Gou et al., 2001; Krackhardt et al., 2002; Sun et al., 2003, 2004, 2006; Scherer et al., 2006; Guo et al., 2006; Shao et al., 2006; Wang et al., 2008, 2012, 2013; Zhu et al., 2008; Chun et al., 2008; Zhao et al., 2008; Zeng et al., 2012; Hu et al., 2013)
Q6P280	**Zinc finger protein 529**	***ZNF529***	May be involved in transcriptional regulation.	(Nagase et al., 2000b; Ota et al., 2004; Grimwood et al., 2004; Gerhard et al., 2004; Hendriks et al., 2017)
Q6P9A1	**Zinc finger protein 530**	***ZNF530***	May be involved in transcriptional regulation.	(Nagase et al., 2000a; Grimwood et al., 2004; Gerhard et al., 2004)
Q6P9A3	**Zinc finger protein 549**	***ZNF549***	May be involved in transcriptional regulation.	(Ota et al., 2004; Grimwood et al., 2004; Gerhard et al., 2004; Hendriks et al., 2017)
Q5MCW4	**Zinc finger protein 569**	***ZNF569***	May be involved in transcriptional regulation.	(Lichter et al., 1992; Ota et al., 2004; Grimwood et al., 2004; Gerhard et al., 2004; Huang et al., 2006; Sjöblom et al., 2006)
Q6ZNG1	**Zinc finger protein 600**	***ZNF600***	May be involved in transcriptional regulation.	(Ota et al., 2004; Grimwood et al., 2004; Bechtel et al., 2007; Mayya et al., 2009)
Q6PG37	**Zinc finger protein 790**	***ZNF790***	May be involved in transcriptional regulation.	(Grimwood et al., 2004; Gerhard et al., 2004)

A8MQ14	**Zinc finger protein 850**	***ZNF850***	May be involved in transcriptional regulation.	(Grimwood et al., 2004)

References

Abrink M, Aveskogh M, Hellman L. 1995. Isolation of cDNA clones for 42 different Krüppel-related zinc finger proteins expressed in the human monoblast cell line U-937. *DNA and cell biology* **14:**125–136. DOI: 10.1089/dna.1995.14.125.

Bechtel S, Rosenfelder H, Duda A, Schmidt CP, Ernst U, Wellenreuther R, Mehrle A, Schuster C, Bahr A, Blöcker H, Heubner D, Hoerlein A, Michel G, Wedler H, Köhrer K, Ottenwälder B, Poustka A, Wiemann S, Schupp I. 2007. The full-ORF clone resource of the German cDNA Consortium. *BMC genomics* **8:**399. DOI: 10.1186/1471-2164-8-399.

Bellefroid EJ, Lecocq PJ, Benhida A, Poncelet DA, Belayew A, Martial JA. 1989. The human genome contains hundreds of genes coding for finger proteins of the Krüppel type. *DNA* **8:**377–387. DOI: 10.1089/dna.1.1989.8.377.

Chun JN, Song IS, Kang D-H, Song HJ, Kim HI, Seo J, Lee K-J, Kim J, Kang SW. 2008. A splice variant of the C(2)H(2)-type zinc finger protein, ZNF268s, regulates NF-kappaB activation by TNF-alpha. *Molecules and cells* **26:**175–180.

Gerhard DS, Wagner L, Feingold EA, Shenmen CM, Grouse LH, Schuler G, Klein SL, Old S, Rasooly R, Good P, Guyer M, Peck AM, Derge JG, Lipman D, Collins FS, Jang W, Sherry S, Feolo M, Misquitta L, Lee E, Rotmistrovsky K, Greenhut SF, Schaefer CF, Buetow K, Bonner TI, Haussler D, Kent J, Kiekhaus M, Furey T, Brent M, Prange C, Schreiber K, Shapiro N, Bhat NK, Hopkins RF, Hsie F, Driscoll T, Soares MB, Casavant TL, Scheetz TE, Brown-stein MJ, Usdin TB, Toshiyuki S, Carninci P, Piao Y, Dudekula DB, Ko MSH, Kawakami K, Suzuki Y, Sugano S, Gruber CE, Smith MR, Simmons B, Moore T, Waterman R, Johnson SL, Ruan Y, Wei CL, Mathavan S, Gunaratne PH, Wu J, Garcia AM, Hulyk SW, Fuh E, Yuan Y, Sneed A, Kowis C, Hodgson A, Muzny DM, McPherson J, Gibbs RA, Fahey J, Helton E, Ketteman M, Madan A, Rodrigues S, Sanchez A, Whiting M, Madari A, Young AC, Wetherby KD, Granite SJ, Kwong PN, Brinkley CP, Pearson RL, Bouffard GG, Blakesly RW, Green ED, Dickson MC, Rodriguez AC, Grimwood J, Schmutz J, Myers RM, Butterfield YSN, Griffith M, Griffith OL, Krzywinski MI, Liao N, Morin R, Palmquist D, Petrescu AS, Skalska U, Smailus DE, Stott JM, Schnerch A, Schein JE, Jones SJM, Holt RA, Baross A, Marra MA, Clifton S, Makowski KA, Bosak S, Malek J, MGC Project Team. 2004. The status, quality, and expansion of the NIH full-length cDNA project: the Mammalian Gene Collection (MGC). *Genome research* **14:**2121–2127. DOI: 10.1101/gr.2596504.

Gou DM, Sun Y, Gao L, Chow LM, Huang J, Feng YD, Jiang DH, Li WX. 2001. Cloning and characterization of a novel Krüppel-like zinc finger gene, ZNF268, expressed in early human embryo. *Biochimica et biophysica acta* **1518:**306–310. DOI: 10.1016/s0167-4781(01)00194-4.

Grimwood J, Gordon LA, Olsen A, Terry A, Schmutz J, Lamerdin J, Hellsten U, Goodstein D, Couronne O, Tran-Gyamfi M, Aerts A, Altherr M, Ashworth L, Bajorek E, Black S, Branscomb E, Caenepeel S, Carrano A, Caoile C, Chan YM, Christensen M, Cleland CA, Copeland A, Dalin E, Dehal P, Denys M, Detter JC, Escobar J, Flowers D, Fotopulos D, Garcia C, Georgescu AM, Glavina T, Gomez M, Gonzales E, Groza M, Hammon N, Hawkins T, Haydu L, Ho I, Huang W, Israni S, Jett J, Kadner K, Kimball H, Kobayashi A, Larionov V, Leem S-H, Lopez F, Lou Y, Lowry S, Malfatti S, Martinez D, McCready P, Medina C, Morgan J, Nelson K, Nolan M, Ovcharenko I, Pitluck S, Pollard M, Popkie AP, Predki P, Quan G, Ramirez L, Rash S, Retterer J, Rodriguez A, Rogers S, Salamov A, Salazar A, She X, Smith D, Slezak T, Solovyev V, Thayer N, Tice H, Tsai M, Ustaszewska A, Vo N, Wagner M, Wheeler J, Wu K, Xie G, Yang J, Dubchak I, Furey TS, DeJong P, Dickson M, Gordon D, Eichler EE, Pennacchio LA, Richardson P, Stubbs L, Rokhsar DS, Myers RM, Rubin EM, Lucas SM. 2004. The DNA sequence and biology of human chromosome 19. *Nature* **428:**529–535. DOI: 10.1038/nature02399.

Guo M-X, Wang D, Shao H-J, Qiu H-L, Xue L, Zhao Z-Z, Zhu C-G, Shi Y-B, Li W-X. 2006. Transcription of human zinc finger ZNF268 gene requires an intragenic promoter element. *The Journal of biological chemistry* **281:**24623–24636. DOI: 10.1074/jbc.M602753200.

Han ZG, Zhang QH, Ye M, Kan LX, Gu BW, He KL, Shi SL, Zhou J, Fu G, Mao M, Chen SJ, Yu L, Chen Z. 1999. Molecular

cloning of six novel Krüppel-like zinc finger genes from hematopoietic cells and identification of a novel transregulatory domain KRNB. *The Journal of biological chemistry* **274:**35741–35748. DOI: 10.1074/jbc.274.50.35741.

Hendriks IA, D'Souza RCJ, Yang B, Verlaan-de Vries M, Mann M, Vertegaal ACO. 2014. Uncovering global SUMOylation signaling networks in a site-specific manner. *Nature structural & molecular biology* **21:**927–936. DOI: 10.1038/nsmb.2890.

Hendriks IA, Lyon D, Young C, Jensen LJ, Vertegaal ACO, Nielsen ML. 2017. Site-specific mapping of the human SUMO proteome reveals co-modification with phosphorylation. *Nature structural & molecular biology* **24:**325–336. DOI: 10.1038/nsmb.3366.

Huang X, Yuan W, Huang W, Bai Y, Deng Y, Zhu C, Liang P, Li Y, Du X, Liu M, Wang Y, Wu X. 2006. ZNF569, a novel KRAB-containing zinc finger protein, suppresses MAPK signaling pathway. *Biochemical and biophysical research communications* **346:**621–628. DOI: 10.1016/j.bbrc.2006.05.109.

Hu L, Wang W, Cai J, Luo J, Huang Y, Xiong S, Li W, Guo M. 2013. Aberrant expression of ZNF268 alters the growth and migration of ovarian cancer cells. *Oncology letters* **6:**49–54. DOI: 10.3892/ol.2013.1318.

Krackhardt AM, Witzens M, Harig S, Hodi FS, Zauls AJ, Chessia M, Barrett P, Gribben JG. 2002. Identification of tumor-associated antigens in chronic lymphocytic leukemia by SEREX. *Blood* **100:**2123–2131. DOI: 10.1182/blood-2002-02-0513.

Lichter P, Bray P, Ried T, Dawid IB, Ward DC. 1992. Clustering of C2-H2 zinc finger motif sequences within telomeric and fragile site regions of human chromosomes. *Genomics* **13:**999–1007. DOI: 10.1016/0888-7543(92)90013-i.

Mayya V, Lundgren DH, Hwang S-I, Rezaul K, Wu L, Eng JK, Rodionov V, Han DK. 2009. Quantitative phosphoproteomic analysis of T cell receptor signaling reveals system-wide modulation of protein-protein interactions. *Science signaling* **2:**ra46. DOI: 10.1126/scisignal.2000007.

Nagase T, Kikuno R, Ishikawa K, Hirosawa M, Ohara O. 2000a. Prediction of the coding sequences of unidentified human genes. XVII. The complete sequences of 100 new cDNA clones from brain which code for large proteins in vitro. *DNA research: an international journal for rapid publication of reports on genes and genomes* **7:**143–150. DOI: 10.1093/dnares/7.2.143.

Nagase T, Kikuno R, Nakayama M, Hirosawa M, Ohara O. 2000b. Prediction of the coding sequences of unidentified human genes. XVIII. The complete sequences of 100 new cDNA clones from brain which code for large proteins in vitro. *DNA research: an international journal for rapid publication of reports on genes and genomes* **7:**273–281. DOI: 10.1093/dnares/7.4.271.

Ota T, Suzuki Y, Nishikawa T, Otsuki T, Sugiyama T, Irie R, Wakamatsu A, Hayashi K, Sato H, Nagai K, Kimura K, Makita H, Sekine M, Obayashi M, Nishi T, Shibahara T, Tanaka T, Ishii S, Yamamoto J-I, Saito K, Kawai Y, Isono Y, Nakamura Y, Nagahari K, Murakami K, Yasuda T, Iwayanagi T, Wagatsuma M, Shiratori A, Sudo H, Hosoiri T, Kaku Y, Kodaira H, Kondo H, Sugawara M, Takahashi M, Kanda K, Yokoi T, Furuya T, Kikkawa E, Omura Y, Abe K, Kamihara K, Katsuta N, Sato K, Tanikawa M, Yamazaki M, Ninomiya K, Ishibashi T, Yamashita H, Murakawa K, Fujimori K, Tanai H, Kimata M, Watanabe M, Hiraoka S, Chiba Y, Ishida S, Ono Y, Takiguchi S, Watanabe S, Yosida M, Hotuta T, Kusano J, Kanehori K, Takahashi-Fujii A, Hara H, Tanase T-O, Nomura Y, Togiya S, Komai F, Hara R, Takeuchi K, Arita M, Imose N, Musashino K, Yuuki H, Oshima A, Sasaki N, Aotsuka S, Yoshikawa Y, Matsunawa H, Ichihara T, Shiohata N, Sano S, Moriya S, Momiyama H, Satoh N, Takami S, Terashima Y, Suzuki O, Nakagawa S, Senoh A, Mizoguchi H, Goto Y, Shimizu F, Wakebe H, Hishigaki H, Watanabe T, Sugiyama A, Takemoto M, Kawakami B, Yamazaki M, Watanabe K, Kumagai A, Itakura S, Fukuzumi Y, Fujimori Y, Komiyama M, Tashiro H, Tanigami A, Fujiwara T, Ono T, Yamada K, Fujii Y, Ozaki K, Hirao M, Ohmori Y, Kawabata A, Hikiji T, Kobatake N, Inagaki H, Ikema Y, Okamoto S, Okitani R, Kawakami T, Noguchi S, Itoh T, Shigeta K, Senba T, Matsumura K, Nakajima Y, Mizuno T, Morinaga M, Sasaki M, Togashi T, Oyama M, Hata H, Watanabe M, Komatsu T, Mizushima-Sugano J, Satoh T, Shirai Y, Takahashi Y, Nakagawa K, Okumura K, Nagase T, Nomura N, Kikuchi H, Masuho Y, Yamashita R, Nakai K, Yada T, Nakamura Y, Ohara O, Isogai T, Sugano S. 2004. Complete sequencing and characterization of 21,243 full-length human cDNAs. *Nature genetics* **36:**40–45. DOI: 10.1038/ng1285.

Rigbolt KTG, Prokhorova TA, Akimov V, Henningsen J, Johansen PT, Kratchmarova I, Kassem M, Mann M, Olsen JV,

Blagoev B. 2011. System-wide temporal characterization of the proteome and phosphoproteome of human embryonic stem cell differentiation. *Science signaling* **4:**rs3. DOI: 10.1126/scisignal.2001570.

Rosati M, Marino M, Franzè A, Tramontano A, Grimaldi G. 1991. Members of the zinc finger protein gene family sharing a conserved N-terminal module. *Nucleic acids research* **19:**5661–5667. DOI: 10.1093/nar/19.20.5661.

Scherer SE, Muzny DM, Buhay CJ, Chen R, Cree A, Ding Y, Dugan-Rocha S, Gill R, Gunaratne P, Harris RA, Hawes AC, Hernandez J, Hodgson AV, Hume J, Jackson A, Khan ZM, Kovar-Smith C, Lewis LR, Lozado RJ, Metzker ML, Milosavljevic A, Miner GR, Montgomery KT, Morgan MB, Nazareth LV, Scott G, Sodergren E, Song X-Z, Steffen D, Lovering RC, Wheeler DA, Worley KC, Yuan Y, Zhang Z, Adams CQ, Ansari-Lari MA, Ayele M, Brown MJ, Chen G, Chen Z, Clerc-Blankenburg KP, Davis C, Delgado O, Dinh HH, Draper H, Gonzalez-Garay ML, Havlak P, Jackson LR, Jacob LS, Kelly SH, Li L, Li Z, Liu J, Liu W, Lu J, Maheshwari M, Nguyen B-V, Okwuonu GO, Pasternak S, Perez LM, Plopper FJH, Santibanez J, Shen H, Tabor PE, Verduzco D, Waldron L, Wang Q, Williams GA, Zhang J, Zhou J, Allen CC, Amin AG, Anyalebechi V, Bailey M, Barbaria JA, Bimage KE, Bryant NP, Burch PE, Burkett CE, Burrell KL, Calderon E, Cardenas V, Carter K, Casias K, Cavazos I, Cavazos SR, Ceasar H, Chacko J, Chan SN, Chavez D, Christopoulos C, Chu J, Cockrell R, Cox CD, Dang M, Dathorne SR, David R, Davis CM, Davy-Carroll L, Deshazo DR, Donlin JE, D'Souza L, Eaves KA, Egan A, Emery-Cohen AJ, Escotto M, Flagg N, Forbes LD, Gabisi AM, Garza M, Hamilton C, Henderson N, Hernandez O, Hines S, Hogues ME, Huang M, Idlebird DG, Johnson R, Jolivet A, Jones S, Kagan R, King LM, Leal B, Lebow H, Lee S, LeVan JM, Lewis LC, London P, Lorensuhewa LM, Loulseged H, Lovett DA, Lucier A, Lucier RL, Ma J, Madu RC, Mapua P, Martindale AD, Martinez E, Massey E, Mawhiney S, Meador MG, Mendez S, Mercado C, Mercado IC, Merritt CE, Miner ZL, Minja E, Mitchell T, Mohabbat F, Mohabbat K, Montgomery B, Moore N, Morris S, Munidasa M, Ngo RN, Nguyen NB, Nickerson E, Nwaokelemeh OO, Nwokenkwo S, Obregon M, Oguh M, Oragunye N, Oviedo RJ, Parish BJ, Parker DN, Parrish J, Parks KL, Paul HA, Payton BA, Perez A, Perrin W, Pickens A, Primus EL, Pu L-L, Puazo M, Quiles MM, Quiroz JB, Rabata D, Reeves K, Ruiz SJ, Shao H, Sisson I, Sonaike T, Sorelle RP, Sutton AE, Svatek AF, Svetz LA, Tamerisa KS, Taylor TR, Teague B, Thomas N, Thorn RD, Trejos ZY, Trevino BK, Ukegbu ON, Urban JB, Vasquez LI, Vera VA, Villasana DM, Wang L, Ward-Moore S, Warren JT, Wei X, White F, Williamson AL, Wleczyk R, Wooden HS, Wooden SH, Yen J, Yoon L, Yoon V, Zorrilla SE, Nelson D, Kucherlapati R, Weinstock G, Gibbs RA, Baylor College of Medicine Human Genome Sequencing Center Sequence Production Team. 2006. The finished DNA sequence of human chromosome 12. *Nature* **440:**346–351. DOI: 10.1038/nature04569.

Shannon M, Hamilton AT, Gordon L, Branscomb E, Stubbs L. 2003. Differential expansion of zinc-finger transcription factor loci in homologous human and mouse gene clusters. *Genome research* **13:**1097–1110. DOI: 10.1101/gr.963903.

Shao H, Zhu C, Zhao Z, Guo M, Qiu H, Liu H, Wang D, Xue L, Gao L, Sun C, Li W. 2006. KRAB-containing zinc finger gene ZNF268 encodes multiple alternatively spliced isoforms that contain transcription regulatory domains. *International journal of molecular medicine* **18:**457–463.

Sjöblom T, Jones S, Wood LD, Parsons DW, Lin J, Barber TD, Mandelker D, Leary RJ, Ptak J, Silliman N, Szabo S, Buckhaults P, Farrell C, Meeh P, Markowitz SD, Willis J, Dawson D, Willson JKV, Gazdar AF, Hartigan J, Wu L, Liu C, Parmigiani G, Park BH, Bachman KE, Papadopoulos N, Vogelstein B, Kinzler KW, Velculescu VE. 2006. The consensus coding sequences of human breast and colorectal cancers. *Science* **314:**268–274. DOI: 10.1126/science.1133427.

Sun Y, Gou D-M, Liu H, Peng X, Li W-X. 2003. The KRAB domain of zinc finger gene ZNF268: a potential transcriptional repressor. *IUBMB life* **55:**127–131. DOI: 10.1080/1521654031000110208.

Sun Y, Shao H, Li Z, Liu J, Gao L, Peng X, Meng Y, Li W. 2004. ZNF268, a novel kruppel-like zinc finger protein, is implicated in early human liver development. *International journal of molecular medicine* **14:**971–975.

Sun C, Zhao Z-Z, Gao L, Sun Y, Shao H-J, Li W-X. 2006. [Cloning and characterization of two novel alternatively spliced transcripts of ZNF268]. *Yi chuan = Hereditas / Zhongguo yi chuan xue hui bian ji* **28:**513–517.

Thiesen HJ. 1990. Multiple genes encoding zinc finger domains are expressed in human T cells. *The New biologist* **2:**363–374.

Tommerup N, Vissing H. 1995. Isolation and fine mapping of 16 novel human zinc finger-encoding cDNAs identify putative candidate genes for developmental and malignant disorders. *Genomics* **27:**259–264. DOI:

10.1006/geno.1995.1040.

Vissing H, Meyer WK, Aagaard L, Tommerup N, Thiesen HJ. 1995. Repression of transcriptional activity by heterologous KRAB domains present in zinc finger proteins. *FEBS letters* **369:**153–157. DOI: 10.1016/0014-5793(95)00728-r.

Wang W, Cai J, Wu Y, Hu L, Chen Z, Hu J, Chen Z, Li W, Guo M, Huang Z. 2013. Novel activity of KRAB domain that functions to reinforce nuclear localization of KRAB-containing zinc finger proteins by interacting with KAP1. *Cellular and molecular life sciences: CMLS* **70:**3947–3958. DOI: 10.1007/s00018-013-1359-4.

Wang W, Guo M, Hu L, Cai J, Zeng Y, Luo J, Shu Z, Li W, Huang Z. 2012. The zinc finger protein ZNF268 is overexpressed in human cervical cancer and contributes to tumorigenesis via enhancing NF-κB signaling. *The Journal of biological chemistry* **287:**42856–42866. DOI: 10.1074/jbc.M112.399923.

Wang D, Guo M-X, Hu H-M, Zhao Z-Z, Qiu H-L, Shao H-J, Zhu C-G, Xue L, Shi Y-B, Li W-X. 2008. Human T-cell leukemia virus type 1 oncoprotein tax represses ZNF268 expression through the cAMP-responsive element-binding protein/activating transcription factor pathway. *The Journal of biological chemistry* **283:**16299–16308. DOI: 10.1074/jbc.M706426200.

Zeng Y, Wang W, Ma J, Wang X, Guo M, Li W. 2012. Knockdown of ZNF268, which is transcriptionally downregulated by GATA-1, promotes proliferation of K562 cells. *PloS one* **7:**e29518. DOI: 10.1371/journal.pone.0029518.

Zhao Z, Wang D, Zhu C, Shao H, Sun C, Qiu H, Xue L, Xu J, Guo M, Li W. 2008. Aberrant alternative splicing of human zinc finger gene ZNF268 in human hematological malignancy. *Oncology reports* **20:**1243–1248.

Zhou H, Di Palma S, Preisinger C, Peng M, Polat AN, Heck AJR, Mohammed S. 2013. Toward a comprehensive characterization of a human cancer cell phosphoproteome. *Journal of proteome research* **12:**260–271. DOI: 10.1021/pr300630k.

Zhu C, Zhao Z, Guo M, Shao H, Qiu H, Wang D, Xu J, Xue L, Li W. 2008. The mammalian gene ZNF268 is regulated by hUpf1. *Biochemistry. Biokhimiia* **73:**881–885. DOI: 10.1134/s0006297908080051.
