# Supplementary material for: Ectopic expression of HIV-1 Tat modifies gene expression in cultured B cells: implications for the development of B-cell lymphomas in HIV-1-infected patients
Source: PeerJ. 2022 Oct 18;10:e13986. doi: 10.7717/peerj.13986 (PMC9586123; doi:10.7717/peerj.13986)
Supplement: Supplemental Information 5 [file peerj-10-13986-s005.pdf]

Table S3

Upregulated and downregulated long non-coding RNAs (PPMI^{Tat} vs RPMI cells).

Upregulated lncRNAs

EnsID	Gene name	Gene type	padj	log2FC	FC	Mean count	Functions and possible involvement in oncogenesis	Reference
ENSG00000264345	LINC01894	lincRNA	1,0E-03	3,35	10,20	8,28	-	
ENSG00000258231	RP11-362K2.2	lincRNA	1,8E-24	2,95	7,74	101,80	Can be part of fusion genes in sarcoma	(Hu et al., 2018b)
ENSG00000255693	RP11-766N7.3	lincRNA	7,5E-13	2,57	5,94	30,74	Acts as tumor suppressor in esophageal squamous cell carcinoma	(Yao et al., 2017)
ENSG00000235385	LINC02154	lincRNA	8,5E-79	2,43	5,40	272,56	Upregulated in Laryngeal Squamous Cell Carcinoma and renal cell carcinoma and can be used as a prognostic feature; promotes the proliferation and metastasis of hepatocellular carcinoma	(Zuo et al., 2018; Gong et al., 2020; Yue et al., 2022)
ENSG00000277448	RP11-538C21.2	lincRNA	5,0E-04	1,87	3,66	12,23	-	
ENSG00000222041	CYTOR	lincRNA	8,8E-03	1,69	3,23	8,01	Involved in tumorigenesis	(Ou et al., 2021)
ENSG00000224559	LINC01087	lincRNA	1,3E-07	1,69	3,23	47,37	Upregulated in breast cancer and thyroid cancer	(She et al., 2020; Yin et al., 2022)
ENSG00000250038	RP11-180C1.1	lincRNA	3,0E-17	1,67	3,19	78,92	-	

EnsID	Gene name	Gene type	padj	log2FC	FC	Mean count	Functions and possible involvement in oncogenesis	Reference
ENSG00000227579	RP1-35C21.2	lincRNA	5,5E-14	1,66	3,16	106,44	Upregulated in NF1/PTPN11 altered vs NF1/PTPN11 WT of histiocytic sarcoma; potential prognostic feature in tongue squamous cell carcinoma	(Egan et al., 2020; Zhang et al., 2020c)
ENSG00000230975	AC084193.1	lincRNA	1,9E-16	1,65	3,14	249,69	-	
ENSG00000253524	RP11-89M20.2	lincRNA	1,4E-02	1,42	2,68	11,67	-	
ENSG00000283408	RP1-242N11.1	lincRNA	2,1E-21	1,39	2,62	174,53	-	
ENSG00000249464	LINC01091	lincRNA	1,7E-02	1,38	2,60	8,30	Upregulated in acute myeloid leukemia	(Hughes et al., 2014)
ENSG00000270069	MIR222HG	lincRNA	5,7E-19	1,36	2,57	205,75	Upregulated in prostate cancer	(Sun et al., 2018)
ENSG00000231877	RP11-177F15.1	lincRNA	2,1E-10	1,36	2,56	97,00	-	
ENSG00000251562	MALAT1	lincRNA	9,9E-22	1,28	2,44	34 783,28		
ENSG00000248869	RP11-138I17.1	lincRNA	4,1E-05	1,28	2,42	42,33	-	
ENSG00000248300	RP11-74M11.2	lincRNA	5,1E-11	1,27	2,41	212,11	-	
ENSG00000280542	AP000432.4	lincRNA	6,5E-04	1,24	2,37	23,94	-	
ENSG00000226785	AC073218.1	lincRNA	7,0E-05	1,22	2,32	39,78	-	
ENSG00000215808	LINC01139	lincRNA	1,6E-18	1,20	2,29	227,87	Upregulated in hepatocellular carcinoma and promotes its progression	(Li et al., 2020)
ENSG00000259348	RP11-4G2.1	lincRNA	1,6E-05	1,20	2,29	38,27	-	
ENSG00000277693	AP003900.6	lincRNA	5,6E-07	1,19	2,29	120,48	Upregulated in oral squamous cell carcinoma and prognostic feature	(Hu et al., 2018a)

EnsID	Gene name	Gene type	padj	log2FC	FC	Mean count	Functions and possible involvement in oncogenesis	Reference
ENSG00000226965	AC003088.1	lincRNA	2,3E-11	1,19	2,29	319,39	Upregulated in breast cancer line	(Li et al., 2021)
ENSG00000249645	RP11-552M14.1	lincRNA	2,0E-22	1,19	2,28	261,05	-	
ENSG00000259761	RP11-138H10.2	lincRNA	7,8E-03	1,14	2,21	20,58	Upregulated in palbociclib-resistant breast cancer cells	(Lanceta et al., 2020)
ENSG00000274090	RP11-415G4.1	lincRNA	2,3E-05	1,03	2,05	92,55	-	
ENSG00000226364	LINC02089	lincRNA	2,7E-02	1,01	2,01	13,51	-	
ENSG00000259527	LINC00052	lincRNA	3,6E-02	1,00	2,00	9,54	Tumorigenesis, progression and metastasis of multiple types of human cancer	(Xiong, Wang & Chen, 2021)
ENSG00000235643	LINC01647	lincRNA	3,5E-02	0,99	1,99	10,16	-	
ENSG00000248837	RP11-412P11.1	lincRNA	9,5E-08	0,99	1,99	859,73	-	
ENSG00000275443	RP11-759A24.3	lincRNA	6,0E-06	0,96	1,95	177,48	-	
ENSG00000234464	RP11-136B18.2	lincRNA	1,7E-02	0,94	1,92	19,24	-	
ENSG00000262202	RP11-160E2.6	lincRNA	4,0E-07	0,94	1,92	94,04	SIRT7 may regulate cell proliferation in breast cancer through this lncRNA	(Chen et al., 2017a)
ENSG00000259702	RP11-236L14.1	lincRNA	5,6E-06	0,93	1,91	70,88	Upregulated in HER2+HR− breast cancer	(Vishnubalaji et al., 2019)
ENSG00000234663	LINC01934	lincRNA	1,4E-09	0,90	1,86	516,91	Downregulated in thyroid carcinoma	(Zhang et al., 2019a)
ENSG00000258131	RP11-541G9.1	lincRNA	2,3E-02	0,89	1,85	14,86	-	
ENSG00000238129	RP3-410C9.2	lincRNA	8,2E-15	0,86	1,82	307,45	-	

EnsID	Gene name	Gene type	padj	log2FC	FC	Mean count	Functions and possible involvement in oncogenesis	Reference
ENSG00000256725	RP11-662M24.2	lincRNA	1,8E-02	0,86	1,81	71,13	-	
ENSG00000256582	RP11-75L1.1	lincRNA	1,3E-02	0,84	1,79	29,52	-	
ENSG00000254456	RP11-405K6.1	lincRNA	2,5E-02	0,81	1,75	20,88	-	
ENSG00000271576	RP11-486G15.2	lincRNA	4,8E-02	0,80	1,74	12,89	Downregulated after Notch2 knockdown in B cell lymphoma; ferroptosis-related gene and prognostic feature in ovarian cancer	(Arasu, Balakrishnan & Velusamy, 2020; Wang et al., 2022)
ENSG00000248457	LINC02220	lincRNA	1,9E-03	0,75	1,68	86,94	-	
ENSG00000245532	NEAT1	lincRNA	2,2E-08	0,73	1,66	3 039,09		
ENSG00000257894	RP1-78O14.1	lincRNA	1,0E-02	0,71	1,63	57,13	Downregulated in lung squamous cell carcinoma	(Chen et al., 2017b)
ENSG00000227863	AC002383.2	lincRNA	6,2E-07	0,68	1,61	200,38	-	
ENSG00000255198	SNHG9	lincRNA	3,4E-03	0,68	1,60	130,94	Upregulated in glioblastoma	(Zhang et al., 2019c)
ENSG00000260658	RP11-368L12.1	lincRNA	7,0E-05	0,67	1,59	1 545,91	Co-expressed with gene module of actively proliferating pre-B cells	(Petri et al., 2015)
ENSG00000258123	RP11-314D7.2	lincRNA	1,7E-03	0,66	1,58	134,22	Differentially expressed in primary vs metastatic sites in HGSC	(Hensler et al., 2020)
ENSG00000259865	RP11-488L18.10	lincRNA	1,3E-02	0,64	1,56	45,36	Potential prognostic biomarker for hepatocellular carcinoma	(Wang et al., 2019b)
ENSG00000226397	C12orf77	lincRNA	3,6E-02	0,62	1,54	46,54	Upregulated in triple negative breast cancer	(Xie et al., 2022)
ENSG00000222042	AP000233.4	lincRNA	5,0E-04	0,60	1,52	155,30	-	

EnsID	Gene name	Gene type	padj	log2FC	FC	Mean count	Functions and possible involvement in oncogenesis	Reference
ENSG00000261474	RP11-452L6.1	lincRNA	5,4E-03	0,60	1,52	63,15	Potential prognostic in clear cell renal cell carcinoma	(Zhang et al., 2019e)
ENSG00000230448	LINC00276	lincRNA	2,6E-04	0,59	1,50	517,66	-	

Downregulated lncRNAs

EnsID	Gene name	Gene type	padj	log2FC	FC	Mean count	Functions and possible involvement in oncogenesis	Reference
ENSG00000231772	RP1-154K9.2	lincRNA	8,3E-53	-9,37	0,00	726,17	-	
ENSG00000224810	RP11-538D16.2	lincRNA	1,9E-10	-9,16	0,00	32,24	Upregulated in hepatocellular carcinoma	(Hou et al., 2018)
ENSG00000233103	RP11-524P6.1	lincRNA	3,2E-07	-7,56	0,01	11,55	-	
ENSG00000235142	RP1-60O19.1	lincRNA	1,9E-38	-7,02	0,01	169,73	Upregulated in gastric cancer	(Zhang et al., 2019b)
ENSG00000218357	LINC01644	lincRNA	1,7E-08	-6,21	0,01	28,17	Upregulated in gastric cancer	(Zhang et al., 2021)
ENSG00000263110	RP11-189E14.3	lincRNA	1,4E-04	-5,92	0,02	5,12	-	
ENSG00000226476	LINC01748	lincRNA	6,9E-20	-5,91	0,02	63,66	Upregulated in NSCLC and many cancers	("lncRNAfunc: Functional analysis of long non-coding RNAs"; Tan et al., 2022)
ENSG00000260058	RP11-279O17.3	lincRNA	1,3E-04	-5,88	0,02	7,00	-	
ENSG00000225179	LINC00457	lincRNA	2,6E-44	-5,62	0,02	139,04	-	

EnsID	Gene name	Gene type	padj	log2FC	FC	Mean count	Functions and possible involvement in oncogenesis	Reference
ENSG00000228918	LINC01344	lincRNA	1,8E-71	-5,55	0,02	262,95	Upregulated in hepatocellular carcinoma	(Esposti et al., 2016)
ENSG00000236938	AC003092.2	lincRNA	6,7E-05	-5,26	0,03	9,82	-	
ENSG00000255621	RP11-377D9.3	lincRNA	4,1E-04	-4,69	0,04	6,44	-	
ENSG00000223850	MYCNUT	lincRNA	1,2E-21	-4,68	0,04	50,08	Upregulated in breast cancer, neuroblastoma; triggers N-Myc RNA upregulation	(Liu et al., 2014; Ravanbakhsh Gavgani et al., 2019)
ENSG00000280870	MIR325HG	lincRNA	1,8E-21	-4,19	0,05	49,17	Upregulated in glioblastoma multiforme	(Xu et al., 2021)
ENSG00000262950	RP11-189E14.5	lincRNA	2,9E-03	-4,02	0,06	4,00	-	
ENSG00000232451	AC016768.1	lincRNA	7,3E-08	-3,62	0,08	13,08	Downregulated in nephroblastoma and liver hepatocellular carcinoma	(“lncRNAfunc: Functional analysis of long non-coding RNAs”; Zhang et al., 2020b)
ENSG00000261319	LINC02152	lincRNA	3,9E-05	-3,56	0,09	10,95	-	
ENSG00000227838	RP1-213J1P__B.1	lincRNA	5,6E-04	-3,44	0,09	8,19	Potential predictive marker for time-to-progression under bevacizumab/erlotinib in advanced non-squamous non-small cell lung cancer	(Baty et al., 2017)
ENSG00000248605	CTD-2306M5.1	lincRNA	2,4E-16	-3,31	0,10	46,00	-	
ENSG00000255364	SMILR	lincRNA	2,3E-04	-3,28	0,10	8,99	Promotes cell proliferation	(Ballantyne et al., 2016)

EnsID	Gene name	Gene type	padj	log2FC	FC	Mean count	Functions and possible involvement in oncogenesis	Reference
ENSG00000260289	CTD-2535I10.1	lincRNA	2,1E-04	-3,27	0,10	10,13	Upregulated in lung adenocarcinoma	(Shi et al., 2018)
ENSG00000267284	RP11-397A16.1	lincRNA	3,1E-07	-3,18	0,11	17,51	Upregulated in HNSCC and lung squamous cell carcinoma	("lncRNAfunc: Functional analysis of long non-coding RNAs"; Nohata, Abba & Gutkind, 2016)
ENSG00000257703	RP11-328J6.1	lincRNA	2,5E-25	-3,18	0,11	72,47	-	
ENSG00000271955	RP11-444A22.1	lincRNA	3,4E-79	-3,03	0,12	803,23	Involved in acute myeloid leukemia cell differentiation	(Cozzi et al., 2022)
ENSG00000242317	RP11-875H7.5	lincRNA	8,7E-12	-2,70	0,15	38,00	Upregulated in lung squamous cell carcinoma	("lncRNAfunc: Functional analysis of long non-coding RNAs")
ENSG00000236453	AC003092.1	lincRNA	2,7E-35	-2,59	0,17	106,23	Downregulated in TMZ resistant glioblastoma cells	(Xu et al., 2018)
ENSG00000254343	RP11-760H22.2	lincRNA	2,5E-06	-2,48	0,18	16,80	Downregulated in esophageal and many other cancers	("lncRNAfunc: Functional analysis of long non-coding RNAs"; Liu et al., 2020)
ENSG00000242268	LINC02082	lincRNA	7,0E-06	-2,37	0,19	23,67	Upregulated in thyroid cancer	(Kim et al., 2016)

EnsID	Gene name	Gene type	padj	log2FC	FC	Mean count	Functions and possible involvement in oncogenesis	Reference
ENSG00000250742	RP11-834C11.4	lincRNA	3,0E-06	-2,25	0,21	21,06	Downregulated in colorectal cancer	(Jafarzadeh et al., 2020)
ENSG00000233005	AC067959.1	lincRNA	1,3E-26	-2,25	0,21	150,71	Upregulated in kidney renal clear cell carcinoma	("lncRNAfunc: Functional analysis of long non-coding RNAs")
ENSG00000229618	AC011288.2	lincRNA	1,6E-15	-2,22	0,22	55,57	Upregulated in many cancers	("lncRNAfunc: Functional analysis of long non-coding RNAs")
ENSG00000249998	RP11-141E13.1	lincRNA	5,6E-03	-2,19	0,22	7,84	-	
ENSG00000254006	RP11-1D12.2	lincRNA	4,3E-36	-2,15	0,22	141,96	-	
ENSG00000255325	RP11-96B2.1	lincRNA	1,1E-02	-2,11	0,23	5,90	-	
ENSG00000259639	RP11-184D12.1	lincRNA	1,7E-05	-2,10	0,23	16,13	-	
ENSG00000243694	LINC02027	lincRNA	4,7E-26	-1,96	0,26	161,99	Downregulated in hepatocellular carcinoma	(Zhang et al., 2017)
ENSG00000224322	AC004009.3	lincRNA	3,1E-07	-1,96	0,26	32,28	-	
ENSG00000231662	RP11-686D16.1	lincRNA	1,3E-03	-1,92	0,26	12,78	-	
ENSG00000185904	LINC00839	lincRNA	2,5E-62	-1,88	0,27	332,95	Downregulated in gastric cancer with H. pilori infection; Upregulated in chemoresistant breast cancer	(Zhong et al., 2018; Chen et al., 2020)
ENSG00000267509	CTD-2043I16.1	lincRNA	2,9E-12	-1,85	0,28	55,45	-	

EnsID	Gene name	Gene type	padj	log2FC	FC	Mean count	Functions and possible involvement in oncogenesis	Reference
ENSG00000231689	LINC01090	lincRNA	4,7E-08	-1,84	0,28	38,40	Potential prognostic feature in lung adenocarcinoma	(Chen et al., 2021)
ENSG00000271860	RP11-436D23.1	lincRNA	1,3E-14	-1,80	0,29	69,51	-	
ENSG00000223351	ZNF385D-AS2	lincRNA	1,7E-02	-1,74	0,30	5,43	Downregulated in hepatocellular carcinoma	(Zhang et al., 2019d)
ENSG00000234056	LINC00463	lincRNA	1,2E-02	-1,71	0,31	6,80	-	
ENSG00000275418	RP11-126O1.6	lincRNA	1,0E-03	-1,64	0,32	15,25	-	
ENSG00000257048	RP11-476M19.2	lincRNA	1,4E-06	-1,56	0,34	32,03	Downregulated in kidney chromophobe	("lncRNAfunc: Functional analysis of long non-coding RNAs")
ENSG00000232006	AC005537.2	lincRNA	1,2E-02	-1,53	0,35	9,39	Potential prognostic feature in pancreatic cancer	(Lei et al., 2021)
ENSG00000237153	RP11-132E11.2	lincRNA	1,1E-03	-1,52	0,35	14,79	-	
ENSG00000254968	RP11-65M17.3	lincRNA	4,8E-02	-1,51	0,35	5,57	Potential prognostic feature in ccRCC	(Zhu et al., 2021)
ENSG00000242741	LINC02005	lincRNA	1,6E-20	-1,44	0,37	324,00	-	
ENSG00000254119	RP11-705O24.1	lincRNA	9,9E-16	-1,40	0,38	1 623,69	Potential prognostic feature in esophageal cancer	(Fan & Liu, 2016)
ENSG00000229719	MIR194-2HG	lincRNA	2,0E-02	-1,37	0,39	8,16	Upregulated in liver cancer and promotes cell proliferation and metastasis	(Xu et al., 2020)
ENSG00000256597	FLJ37505	lincRNA	4,3E-02	-1,36	0,39	5,55	Downregulated in bladder cancer	(Xiang et al., 2020)

EnsID	Gene name	Gene type	padj	log2FC	FC	Mean count	Functions and possible involvement in oncogenesis	Reference
ENSG00000233154	LINC01762	lincRNA	5,4E-09	-1,36	0,39	145,26	Upregulated in esophageal squamous cell carcinoma	(Yao et al., 2016)
ENSG00000283052	RP11-210L7.3	lincRNA	2,6E-07	-1,33	0,40	76,20	-	
ENSG00000225519	RP11-236B18.2	lincRNA	2,2E-11	-1,32	0,40	78,51	-	
ENSG00000225489	RP11-390F4.3	lincRNA	7,6E-04	-1,28	0,41	27,79	Upregulated in colon cancer; promotes metastasis	(Peng et al., 2020; Zhang et al., 2022)
ENSG00000257759	RP11-486O13.4	lincRNA	1,2E-02	-1,27	0,42	19,19	-	
ENSG00000266088	RP5-1028K7.2	lincRNA	8,0E-03	-1,24	0,42	12,64	Potential prognostic feature in breast cancer; upregulated in lung adenocarcinoma	(“lncRNAfunc: Functional analysis of long non-coding RNAs”; Wang et al., 2018)
ENSG00000176659	C20orf197	lincRNA	1,9E-22	-1,23	0,43	217,05	Upregulated in lung adenocarcinoma; downregulated in NSCLC	(“lncRNAfunc: Functional analysis of long non-coding RNAs”; Song et al., 2020)
ENSG00000225546	LVCAT5	lincRNA	2,2E-07	-1,21	0,43	100,32	-	
ENSG00000242759	LINC00882	lincRNA	3,8E-08	-1,19	0,44	103,40	Upregulated in hepatocellular carcinoma	(Zhu et al., 2018)
ENSG00000258667	HIF1A-AS2	lincRNA	5,5E-04	-1,17	0,44	29,81	Upregulated in many cancers	(Chen et al., 2015, 2019; Wang et al., 2019a)

EnsID	Gene name	Gene type	padj	log2FC	FC	Mean count	Functions and possible involvement in oncogenesis	Reference
ENSG00000227681	RP11-307P5.1	lincRNA	4,5E-10	-1,17	0,44	621,53	-	
ENSG00000267327	CTD-2008L17.1	lincRNA	3,8E-05	-1,15	0,45	54,04	-	
ENSG00000234275	AC017053.1	lincRNA	2,9E-02	-1,14	0,46	11,69	-	
ENSG00000267761	CTD-2130O13.1	lincRNA	4,5E-08	-1,13	0,46	895,69	Potential cancer-specific biomarker	(Mohammed et al., 2017)
ENSG00000260725	AC005307.1	lincRNA	2,0E-04	-1,09	0,47	39,22	-	
ENSG00000253103	LINC01609	lincRNA	4,1E-02	-1,07	0,48	8,59	-	
ENSG00000250775	RP11-12K22.1	lincRNA	1,4E-08	-1,07	0,48	108,26	-	
ENSG00000189229	AC069277.2	lincRNA	2,7E-03	-1,06	0,48	21,55	-	
ENSG00000233067	PTCHD1-AS	lincRNA	3,3E-09	-1,01	0,50	198,93	Upregulated in glioma	(Sun et al., 2020)
ENSG00000260073	RP11-488I20.9	lincRNA	2,9E-02	-1,00	0,50	13,36	Potential prognostic feature in esophageal squamous cell carcinoma	(Zhang et al., 2020a)
ENSG00000224565	LINC01754	lincRNA	5,7E-05	-0,99	0,50	53,00	-	
ENSG00000232053	AC009784.3	lincRNA	1,0E-05	-0,98	0,51	125,07	-	
ENSG00000251432	RP11-420A23.1	lincRNA	1,6E-07	-0,98	0,51	237,69	Upregulated in cholangiocarcinoma, kidney renal clear cell carcinoma, kidney renal papillary cell carcinoma	(“lncRNAfunc: Functional analysis of long non-coding RNAs”)
ENSG00000238121	LINC00426	lincRNA	1,1E-07	-0,98	0,51	104,79	Downregulated in lung cancer	(Du et al., 2020)
ENSG00000258958	RP11-594C13.2	lincRNA	4,2E-03	-0,96	0,51	28,86	-	
ENSG00000234807	LINC01135	lincRNA	2,4E-02	-0,95	0,52	16,01	Downregulated in thyroid cancer	(“lncRNAfunc:

EnsID	Gene name	Gene type	padj	log2FC	FC	Mean count	Functions and possible involvement in oncogenesis	Reference
								Functional analysis of long non-coding RNAs”)
ENSG00000258479	LINC00640	lincRNA	4,5E-02	-0,94	0,52	9,39	-	
ENSG00000258760	CTD-2509G16.5	lincRNA	7,0E-06	-0,93	0,52	97,90	-	
ENSG00000260448	LCMT1-AS1	lincRNA	4,2E-03	-0,91	0,53	28,09	Downregulated in colon cancer	(Liu et al., 2022)
ENSG00000230131	RP11-282I1.1	lincRNA	6,3E-03	-0,91	0,53	33,95	-	
ENSG00000235823	OLMALINC	lincRNA	5,0E-12	-0,90	0,53	220,16	Prognostic signature in osteosarcoma	(He et al., 2022)
ENSG00000253377	RP11-566H8.3	lincRNA	6,4E-07	-0,86	0,55	1 751,51	Produces non-canonical cancer/testis antigen	(Chong et al., 2020)
ENSG00000229981	LINC01435	lincRNA	1,4E-04	-0,83	0,56	177,66	-	
ENSG00000231671	LINC01307	lincRNA	9,2E-04	-0,81	0,57	52,73	Upregulated in fibrolamellar hepatocellular carcinoma	(Farber et al., 2018)
ENSG00000267731	RP11-147L13.8	lincRNA	6,8E-03	-0,80	0,57	32,61	Downregulated in gallbladder cancer	(Zheng et al., 2021)
ENSG00000257747	RP11-362A1.1	lincRNA	1,9E-02	-0,78	0,58	29,35	-	
ENSG00000230107	CTA-126B4.7	lincRNA	1,8E-03	-0,76	0,59	50,60	Upregulated in many cancers	(“lncRNAfunc: Functional analysis of long non-coding RNAs”)
ENSG00000258602	LINC01629	lincRNA	1,1E-02	-0,74	0,60	38,01	Upregulated in endometrial cancer	(Yang, Ma & Ma, 2021)

EnsID	Gene name	Gene type	padj	log2FC	FC	Mean count	Functions and possible involvement in oncogenesis	Reference
ENSG00000236081	ELFN1-AS1	lincRNA	4,9E-02	-0,70	0,62	20,00	Upregulated in colon and colorectal cancers	(Dong et al., 2019; Lei, Feng & Hong, 2020)
ENSG00000251381	LINC00958	lincRNA	9,4E-23	-0,68	0,62	1 003,09	Canonical lncRNA in human cancer progression (overexpressed in many cancers)	(Li et al., 2022)
ENSG00000228222	AC074363.1	lincRNA	1,8E-02	-0,67	0,63	40,79	-	
ENSG00000247982	LINC00926	lincRNA	1,5E-02	-0,67	0,63	38,68	Negatively regulates PGK1 expression and predicts good clinical outcome of breast cancer	(Chu et al., 2021)
ENSG00000243479	MNX1-AS1	lincRNA	1,6E-02	-0,63	0,64	44,42	Upregulated in many cancers	(Chu et al., 2019; Shuai et al., 2020; Wang et al., 2020)
ENSG00000233928	RP11-305F18.1	lincRNA	1,1E-02	-0,63	0,65	63,36	-	
ENSG00000251088	RP11-325B23.2	lincRNA	1,5E-04	-0,63	0,65	454,27	-	
ENSG00000283554	RP11-413N19.2	lincRNA	4,2E-02	-0,63	0,65	28,11	-	
ENSG00000235947	EGOT	lincRNA	7,1E-03	-0,59	0,66	60,29	Dysregulated in many cancers	(Du et al., 2013; Wu et al., 2019)

References

Arasu A, Balakrishnan P, Velusamy T. 2020. RNA sequencing analyses reveal differentially expressed genes and pathways as Notch2 targets in B-cell lymphoma. *Oncotarget* 11:4527–4540. DOI: 10.18632/oncotarget.27805.

Ballantyne MD, Pinel K, Dakin R, Vesey AT, Diver L, Mackenzie R, Garcia R, Welsh P, Sattar N, Hamilton G, Joshi N, Dweck MR, Miano JM, McBride MW, Newby DE, McDonald RA, Baker AH. 2016. Smooth Muscle Enriched Long Noncoding RNA (SMILR) Regulates Cell Proliferation. *Circulation* 133:2050–2065. DOI: 10.1161/CIRCULATIONAHA.115.021019.

Baty F, Joerger M, Früh M, Klingbiel D, Zappa F, Brutsche M. 2017. 24h-gene variation effect of combined bevacizumab/erlotinib in advanced non-squamous non-small cell

lung cancer using exon array blood profiling. *Journal of translational medicine* 15:66. DOI: 10.1186/s12967-017-1174-z.

Chen W-M, Huang M, Kong R, Xu T-P, Zhang E-B, Xia R, Sun M, De W, Shu Y-Q. 2015. Antisense Long Noncoding RNA HIF1A-AS2 Is Upregulated in Gastric Cancer and Associated with Poor Prognosis. *Digestive diseases and sciences* 60:1655–1662. DOI: 10.1007/s10620-015-3524-0.

Chen X, Liu M, Meng F, Sun B, Jin X, Jia C. 2019. The long noncoding RNA HIF1A-AS2 facilitates cisplatin resistance in bladder cancer. *Journal of cellular biochemistry* 120:243–252. DOI: 10.1002/jcb.27327.

Chen K-L, Li L, Wang Y-R, Li C-M, Badri TM, Wang G-L. 2017a. Long noncoding RNA and mRNA profiling in MDA-MB-231 cells following RNAi-mediated knockdown of SIRT7. *OncoTargets and therapy* 10:5115–5128. DOI: 10.2147/OTT.S149048.

Chen Q, Shen H, Zhu X, Liu Y, Yang H, Chen H, Xiong S, Chi H, Xu W. 2020. A nuclear lncRNA Linc00839 as a Myc target to promote breast cancer chemoresistance via PI3K/AKT signaling pathway. *Cancer science* 111:3279–3291. DOI: 10.1111/cas.14555.

Chen W-J, Tang R-X, He R-Q, Li D-Y, Liang L, Zeng J-H, Hu X-H, Ma J, Li S-K, Chen G. 2017b. Clinical roles of the aberrantly expressed lncRNAs in lung squamous cell carcinoma: a study based on RNA-sequencing and microarray data mining. *Oncotarget* 8:61282–61304. DOI: 10.18632/oncotarget.18058.

Chen Y, Zhang X, Li J, Zhou M. 2021. Immune-related eight-lncRNA signature for improving prognosis prediction of lung adenocarcinoma. *Journal of clinical laboratory analysis* 35:e24018. DOI: 10.1002/jcla.24018.

Chong C, Müller M, Pak H, Harnett D, Huber F, Grun D, Leleu M, Auger A, Arnaud M, Stevenson BJ, Michaux J, Bilic I, Hirsekorn A, Calviello L, Simó-Riudalbas L, Planet E, Lubiński J, Bryśkiewicz M, Wiznerowicz M, Xenarios I, Zhang L, Trono D, Harari A, Ohler U, Coukos G, Bassani-Sternberg M. 2020. Integrated proteogenomic deep sequencing and analytics accurately identify non-canonical peptides in tumor immunopeptidomes. *Nature communications* 11:1293. DOI: 10.1038/s41467-020-14968-9.

Chu Z, Huo N, Zhu X, Liu H, Cong R, Ma L, Kang X, Xue C, Li J, Li Q, You H, Zhang Q, Xu X. 2021. FOXO3A-induced LINC00926 suppresses breast tumor growth and metastasis through inhibition of PGK1-mediated Warburg effect. *Molecular therapy: the journal of the American Society of Gene Therapy* 29:2737–2753. DOI: 10.1016/j.ymthe.2021.04.036.

Chu J, Li H, Xing Y, Jia J, Sheng J, Yang L, Sun K, Qu Y, Zhang Y, Yin H, Wan J, He F. 2019. LncRNA MNX1-AS1 promotes progression of esophageal squamous cell carcinoma by regulating miR-34a/SIRT1 axis. *Biomedicine & pharmacotherapy = Biomedecine & pharmacotherapie* 116:109029. DOI: 10.1016/j.biopha.2019.109029.

Cozzi E, Neddermeyer A, Miliara S, Lennartsson A, Lehmann S. 2022. Identification of long non-coding RNAs involved in leukemogenesis and venetoclax response in acute myeloid leukemia through functional CRISPR-dCas9 interference screens.

Dong L, Ding C, Zheng T, Pu Y, Liu J, Zhang W, Xue F, Kang P, Ma Y, Wang X, Mao C. 2019. Extracellular vesicles from human umbilical cord mesenchymal stem cells treated with siRNA against ELFN1-AS1 suppress colon adenocarcinoma proliferation and migration. *American journal of translational research* 11:6989–6999.

Du Z, Fei T, Verhaak RGW, Su Z, Zhang Y, Brown M, Chen Y, Liu XS. 2013. Integrative genomic analyses reveal clinically relevant long noncoding RNAs in human cancer. *Nature structural & molecular biology* 20:908–913. DOI: 10.1038/nsmb.2591.

Du W, Sun J, Gu J, Zhang S, Zhang T. 2020. Bioinformatics analysis of LINC00426 expression in lung cancer and its correlation with patients' prognosis. *Thoracic cancer* 11:150–155. DOI: 10.1111/1759-7714.13228.

Egan C, Nicolae A, Lack J, Chung H-J, Skarshaug S, Pham TA, Navarro W, Abdullaev Z, Aguilera NS, Xi L, Pack S, Pittaluga S, Jaffe ES, Raffeld M. 2020. Genomic profiling of primary histiocytic sarcoma reveals two molecular subgroups. *Haematologica* 105:951–960. DOI: 10.3324/haematol.2019.230375.

Esposti DD, Hernandez-Vargas H, Voegele C, Fernandez-Jimenez N, Forey N, Bancel B, Le Calvez-Kelm F, McKay J, Merle P, Herceg Z. 2016. Identification of novel long non-

coding RNAs deregulated in hepatocellular carcinoma using RNA-sequencing. *Oncotarget* 7:31862–31877. DOI: 10.18632/oncotarget.7364.

Fan Q, Liu B. 2016. Identification of a RNA-Seq Based 8-Long Non-Coding RNA Signature Predicting Survival in Esophageal Cancer. *Medical science monitor: international medical journal of experimental and clinical research* 22:5163–5172. DOI: 10.12659/msm.902615.

Farber BA, Lalazar G, Simon EP, Hammond WJ, Requena D, Bhanot UK, La Quaglia MP, Simon SM. 2018. Non coding RNA analysis in fibrolamellar hepatocellular carcinoma. *Oncotarget* 9:10211–10227. DOI: 10.18632/oncotarget.23325.

Gong S, Xu M, Zhang Y, Shan Y, Zhang H. 2020. The Prognostic Signature and Potential Target Genes of Six Long Non-coding RNA in Laryngeal Squamous Cell Carcinoma. *Frontiers in genetics* 11:413. DOI: 10.3389/fgene.2020.00413.

Hensler M, Kasikova L, Fiser K, Rakova J, Skapa P, Laco J, Lanickova T, Pecen L, Truxova I, Vosahlikova S, Moserova I, Praznovec I, Drochytek V, Rehackova M, Brtnicky T, Rob L, Benes V, Pistolic J, Sojka L, Ryska A, Sautes-Fridman C, Fridman WH, Galluzzi L, Spisek R, Fucikova J. 2020. M2-like macrophages dictate clinically relevant immunosuppression in metastatic ovarian cancer. *Journal for immunotherapy of cancer* 8. DOI: 10.1136/jitc-2020-000979.

He Y, Zhou H, Xu H, You H, Cheng H. 2022. Construction of an Immune-Related lncRNA Signature That Predicts Prognosis and Immune Microenvironment in Osteosarcoma Patients. *Frontiers in oncology* 12:769202. DOI: 10.3389/fonc.2022.769202.

Hou Y, Yu Z, Tam NL, Huang S, Sun C, Wang R, Zhang X, Wang Z, Ma Y, He X, Wu L. 2018. Exosome-related lncRNAs as predictors of HCC patient survival: a prognostic model. *American journal of translational research* 10:1648–1662.

Hughes JM, Salvatori B, Giorgi FM, Bozzoni I, Fatica A. 2014. CEBPA-regulated lncRNAs, new players in the study of acute myeloid leukemia. *Journal of hematology & oncology* 7:69. DOI: 10.1186/s13045-014-0069-1.

Hu X, Qiu Z, Zeng J, Xiao T, Ke Z, Lyu H. 2018a. A novel long non-coding RNA, AC012456.4, as a valuable and independent prognostic biomarker of survival in oral squamous cell carcinoma. *PeerJ* 6:e5307. DOI: 10.7717/peerj.5307.

Hu X, Wang Q, Tang M, Barthel F, Amin S, Yoshihara K, Lang FM, Martinez-Ledesma E, Lee SH, Zheng S, Verhaak RGW. 2018b. TumorFusions: an integrative resource for cancer-associated transcript fusions. *Nucleic acids research* 46:D1144–D1149. DOI: 10.1093/nar/gkx1018.

Jafarzadeh M, Soltani BM, Soleimani M, Hosseinkhani S. 2020. Epigenetically silenced LINC02381 functions as a tumor suppressor by regulating PI3K-Akt signaling pathway. *Biochimie* 171-172:63–71. DOI: 10.1016/j.biochi.2020.02.009.

Kim D, Lee WK, Jeong S, Seol M-Y, Kim H, Kim K-S, Lee EJ, Lee J, Jo YS. 2016. Upregulation of long noncoding RNA LOC100507661 promotes tumor aggressiveness in thyroid cancer. *Molecular and cellular endocrinology* 431:36–45. DOI: 10.1016/j.mce.2016.05.002.

Lanceta L, O’Neill C, Lypova N, Li X, Rouchka E, Waigel S, Gomez-Gutierrez JG, Chesney J, Imbert-Fernandez Y. 2020. Transcriptomic Profiling Identifies Differentially Expressed Genes in Palbociclib-Resistant ER+ MCF7 Breast Cancer Cells. *Genes* 11. DOI: 10.3390/genes11040467.

Lei R, Feng L, Hong D. 2020. ELFN1-AS1 accelerates the proliferation and migration of colorectal cancer via regulation of miR-4644/TRIM44 axis. *Cancer biomarkers: section A of Disease markers* 27:433–443. DOI: 10.3233/CBM-190559.

Lei Y, Tang R, Xu J, Zhang B, Liu J, Liang C, Meng Q, Hua J, Yu X, Wang W, Shi S. 2021. Construction of a novel risk model based on the random forest algorithm to distinguish pancreatic cancers with different prognoses and immune microenvironment features. *Bioengineered* 12:3593–3602. DOI: 10.1080/21655979.2021.1951527.

Li H, An X, Li Q, Yu H, Li Z. 2021. Construction and analysis of competing endogenous RNA network of MCF-7 breast cancer cells based on the inhibitory effect of 6-thioguanine on cell proliferation. *Oncology letters* 21:104. DOI: 10.3892/ol.2020.12365.

Li Z-B, Chu H-T, Jia M, Li L. 2020. Long noncoding RNA LINC01139 promotes the progression of hepatocellular carcinoma by upregulating MYBL2 via competitively binding

to miR-30 family. *Biochemical and biophysical research communications* 525:581–588. DOI: 10.1016/j.bbrc.2020.02.116.

Li H, Gao J, Liu L, Zhang S. 2022. LINC00958: A promising long non-coding RNA related to cancer. *Biomedicine & pharmacotherapy = Biomedecine & pharmacotherapie* 151:113087. DOI: 10.1016/j.biopha.2022.113087.

Liu PY, Erriquez D, Marshall GM, Tee AE, Polly P, Wong M, Liu B, Bell JL, Zhang XD, Milazzo G, Cheung BB, Fox A, Swarbrick A, Hüttelmaier S, Kavallaris M, Perini G, Mattick JS, Dinger ME, Liu T. 2014. Effects of a novel long noncoding RNA, lncUSMycN, on N-Myc expression and neuroblastoma progression. *Journal of the National Cancer Institute* 106. DOI: 10.1093/jnci/dju113.

Liu L, Huang L, Chen W, Zhang G, Li Y, Wu Y, Xiong J, Jie Z. 2022. Comprehensive Analysis of Necroptosis-Related Long Noncoding RNA Immune Infiltration and Prediction of Prognosis in Patients With Colon Cancer. *Frontiers in molecular biosciences* 9:811269. DOI: 10.3389/fmolb.2022.811269.

Liu H, Zhang Q, Lou Q, Zhang X, Cui Y, Wang P, Yang F, Wu F, Wang J, Fan T, Li S. 2020. Differential Analysis of lncRNA, miRNA and mRNA Expression Profiles and the Prognostic Value of lncRNA in Esophageal Cancer. *Pathology oncology research: POR* 26:1029–1039. DOI: 10.1007/s12253-019-00655-8.

lncRNAfunc: Functional analysis of long non-coding RNAs. *Available at https://ccsm.uth.edu/lncRNAfunc*

Mohammed A, Biegert G, Adamec J, Helikar T. 2017. Identification of potential tissue-specific cancer biomarkers and development of cancer versus normal genomic classifiers. *Oncotarget* 8:85692–85715. DOI: 10.18632/oncotarget.21127.

Nohata N, Abba MC, Gutkind JS. 2016. Unraveling the oral cancer lncRNAome: Identification of novel lncRNAs associated with malignant progression and HPV infection. *Oral oncology* 59:58–66. DOI: 10.1016/j.oraloncology.2016.05.014.

Ou C, He X, Liu Y, Zhang X. 2021. lncRNA cytoskeleton regulator RNA (CYTOR): Diverse functions in metabolism, inflammation and tumorigenesis, and potential applications in precision oncology. *Genes & Diseases*. DOI: 10.1016/j.gendis.2021.08.012.

Peng P-H, Chieh-Yu Lai J, Hsu K-W, Wu K-J. 2020. Hypoxia-induced lncRNA RP11-390F4.3 promotes epithelial-mesenchymal transition (EMT) and metastasis through upregulating EMT regulators. *Cancer letters* 483:35–45. DOI: 10.1016/j.canlet.2020.04.014.

Petri A, Dybkær K, Bøgsted M, Thrue CA, Hagedorn PH, Schmitz A, Bødker JS, Johnsen HE, Kauppinen S. 2015. Long Noncoding RNA Expression during Human B-Cell Development. *PloS one* 10:e0138236. DOI: 10.1371/journal.pone.0138236.

Ravanbakhsh Gavgani R, Babaei E, Hosseinpourfeizi MA, Fakhrjou A, Montazeri V. 2019. Clinical and in vitro Study of Novel Long Non-Coding RNA lncUSMycN in Breast Cancer. *Iranian biomedical journal* 23:303–311. DOI: 10.29252/.23.5.303.

She J-K, Fu D-N, Zhen D, Gong G-H, Zhang B. 2020. LINC01087 is Highly Expressed in Breast Cancer and Regulates the Malignant Behavior of Cancer Cells Through miR-335-5p/Rock1. *OncoTargets and therapy* 13:9771–9783. DOI: 10.2147/OTT.S255994.

Shi X, Tan H, Le X, Xian H, Li X, Huang K, Luo VY, Liu Y, Wu Z, Mo H, Chen AM, Liang Y, Zhang J. 2018. An expression signature model to predict lung adenocarcinoma-specific survival. *Cancer management and research* 10:3717–3732. DOI: 10.2147/CMAR.S159563.

Shuai Y, Ma Z, Liu W, Yu T, Yan C, Jiang H, Tian S, Xu T, Shu Y. 2020. TEAD4 modulated LncRNA MNX1-AS1 contributes to gastric cancer progression partly through suppressing BTG2 and activating BCL2. *Molecular cancer* 19:6. DOI: 10.1186/s12943-019-1104-1.

Song J-Y, Li X-P, Qin X-J, Zhang J-D, Zhao J-Y, Wang R. 2020. A fourteen-lncRNA risk score system for prognostic prediction of patients with non-small cell lung cancer. *Cancer biomarkers: section A of Disease markers* 29:493–508. DOI: 10.3233/CBM-190505.

Sun T, Du S-Y, Armenia J, Qu F, Fan J, Wang X, Fei T, Komura K, Liu SX, Lee G-SM, Kantoff PW. 2018. Expression of lncRNA MIR222HG co-transcribed from the miR-221/222 gene promoter facilitates the development of castration-resistant prostate cancer. *Oncogenesis* 7:30. DOI: 10.1038/s41389-018-0039-5.

Sun J, Jiang R, Song M, Yao J, Hou S, Zhu Y, Ji X, Sheng H, Tang Z, Liu Q, Jia Z, Shi W, Shi J. 2020. Pathological Grade-Associated Transcriptome Profiling of lncRNAs and mRNAs in Gliomas. *Frontiers in oncology* 10:253. DOI: 10.3389/fonc.2020.00253.

Tan Y, Xu F, Xu L, Cui J. 2022. Long non‑coding RNA LINC01748 exerts carcinogenic effects in non‑small cell lung cancer cell lines by regulating the microRNA‑520a‑5p/HMGA1 axis. *International journal of molecular medicine* 49. DOI: 10.3892/ijmm.2021.5077.

Vishnubalaji R, Shaath H, Elkord E, Alajez NM. 2019. Long non-coding RNA (lncRNA) transcriptional landscape in breast cancer identifies LINC01614 as non-favorable prognostic biomarker regulated by TGFβ and focal adhesion kinase (FAK) signaling. *Cell death discovery* 5:109. DOI: 10.1038/s41420-019-0190-6.

Wang F, Dohogne Z, Yang J, Liu Y, Soibam B. 2018. Predictors of breast cancer cell types and their prognostic power in breast cancer patients. *BMC genomics* 19:137. DOI: 10.1186/s12864-018-4527-y.

Wang K, Mei S, Cai M, Zhai D, Zhang D, Yu J, Ni Z, Yu C. 2022. Ferroptosis-Related Long Noncoding RNAs as Prognostic Biomarkers for Ovarian Cancer. *Frontiers in oncology* 12:888699. DOI: 10.3389/fonc.2022.888699.

Wang X, Peng L, Gong X, Zhang X, Sun R. 2019a. LncRNA HIF1A-AS2 promotes osteosarcoma progression by acting as a sponge of miR-129-5p. *Aging* 11:11803–11813. DOI: 10.18632/aging.102448.

Wang J, Pu J, Yao T, Lu X, Deng Y. 2019b. Four long noncoding RNAs as potential prognostic biomarkers for hepatocellular carcinoma. *Journal of cellular physiology* 234:8709–8716. DOI: 10.1002/jcp.27530.

Wang J, Xing H, Nikzad AA, Liu B, Zhang Y, Li S, Zhang E, Jia Z. 2020. Long Noncoding RNA MNX1 antisense RNA 1 Exerts Oncogenic Functions in Bladder Cancer by Regulating miR-218-5p/RAB1A Axis. *The Journal of pharmacology and experimental therapeutics* 372:237–247. DOI: 10.1124/jpet.119.262949.

Wu S, Ai H, Zhang K, Yun H, Xie F. 2019. Long Non-Coding RNA EGOT Promotes the Malignant Phenotypes of Hepatocellular Carcinoma Cells and Increases the Expression of HMGA2 via Down-Regulating miR-33a-5p. *OncoTargets and therapy* 12:11623–11635. DOI: 10.2147/OTT.S218308.

Xiang H, Liu Z, Zhou YB, Yao Q, Jin L, Xue BX. 2020. [Effects of long non-coding RNA FLJ37505 on the proliferation and migration of bladder cancer cells]. *Zhonghua yi xue za zhi* 100:1249–1254. DOI: 10.3760/cma.j.cn112137-20190728-01676.

Xie J, Tian W, Tang Y, Zou Y, Zheng S, Wu L, Zeng Y, Wu S, Xie X, Xie X. 2022. Establishment of a Cell Necroptosis Index to Predict Prognosis and Drug Sensitivity for Patients With Triple-Negative Breast Cancer. *Frontiers in molecular biosciences* 9:834593. DOI: 10.3389/fmolb.2022.834593.

Xiong D, Wang D, Chen Y. 2021. Role of the long non-coding RNA LINC00052 in tumors. *Oncology letters* 21:316. DOI: 10.3892/ol.2021.12577.

Xu K, Cai Y, Zhang M, Zou H, Chang Z, Li D, Bai J, Xu J, Li Y. 2021. Pan-cancer characterization of expression and clinical relevance of m6A-related tissue-elevated long non-coding RNAs. *Molecular cancer* 20:31. DOI: 10.1186/s12943-021-01324-8.

Xu N, Liu B, Lian C, Doycheva DM, Fu Z, Liu Y, Zhou J, He Z, Yang Z, Huang Q, Zeng H, Guo H. 2018. Long noncoding RNA AC003092.1 promotes temozolomide chemosensitivity through miR-195/TFPI-2 signaling modulation in glioblastoma. *Cell death & disease* 9:1139. DOI: 10.1038/s41419-018-1183-8.

Xu G, Zhu Y, Liu H, Liu Y, Zhang X. 2020. LncRNA MIR194-2HG Promotes Cell Proliferation and Metastasis via Regulation of miR-1207-5p/TCF19/Wnt/β-Catenin Signaling in Liver Cancer. *OncoTargets and therapy* 13:9887–9899. DOI: 10.2147/OTT.S264614.

Yang D, Ma J, Ma X-X. 2021. CDKN2B-AS1 Promotes Malignancy as a Novel Prognosis-Related Molecular Marker in the Endometrial Cancer Immune Microenvironment. *Frontiers in cell and developmental biology* 9:721676. DOI: 10.3389/fcell.2021.721676.

Yao J, Huang J-X, Lin M, Wu Z-D, Yu H, Wang P-C, Ye J, Chen P, Wu J, Zhao G-J. 2016. Microarray expression profile analysis of aberrant long non-coding RNAs in esophageal squamous cell carcinoma. *International journal of oncology* 48:2543–2557. DOI: 10.3892/ijo.2016.3457.

Yao G-L, Pan C-F, Xu H, Wei K, Liu B, Zhai R, Chen Y-J. 2017. Long noncoding RNA RP11-766N7.4 functions as a tumor suppressor by regulating epithelial-mesenchymal transition in esophageal squamous cell carcinoma. *Biomedicine & pharmacotherapy = Biomedecine & pharmacotherapie* 88:778–785. DOI: 10.1016/j.biopha.2017.01.124.

Yin Y, Huang J, Shi H, Huang Y, Huang Z, Song M, Yin L. 2022. LINC01087 Promotes the Proliferation, Migration, and Invasion of Thyroid Cancer Cells by Upregulating PPM1E. *Journal of oncology* 2022:7787378. DOI: 10.1155/2022/7787378.

Yue H, Wu K, Liu K, Gou L, Huang A, Tang H. 2022. LINC02154 promotes the proliferation and metastasis of hepatocellular carcinoma by enhancing SPC24 promoter activity and activating the PI3K-AKT signaling pathway. *Cellular oncology* 45:447–462. DOI: 10.1007/s13402-022-00676-7.

Zhang Y, Jin T, Shen H, Yan J, Guan M, Jin X. 2019a. Identification of Long Non-Coding RNA Expression Profiles and Co-Expression Genes in Thyroid Carcinoma Based on The Cancer Genome Atlas (TCGA) Database. *Medical science monitor: international medical journal of experimental and clinical research* 25:9752–9769. DOI: 10.12659/MSM.917845.

Zhang S, Li S, Guo J-L, Li N, Zhang C-N, Liu J. 2021. Integrated Analysis of lncRNA-Associated ceRNA Network Identifies Two lncRNA Signatures as a Prognostic Biomarker in Gastric Cancer. *Disease markers* 2021:8886897. DOI: 10.1155/2021/8886897.

Zhang L, Li P, Liu E, Xing C, Zhu D, Zhang J, Wang W, Jiang G. 2020a. Prognostic value of a five-lncRNA signature in esophageal squamous cell carcinoma. *Cancer cell international* 20:386. DOI: 10.1186/s12935-020-01480-9.

Zhang S-G, Li Y-F, Zhao N-N, Lai C-C, Cheng S-J, Yan J, Zhang P, Wang Z, Wang X-L, Yang P-H. 2017. Decreased expression of long non-coding RNA LOC728290 in human hepatocellular carcinoma. *Oncology letters* 14:4551–4556. DOI: 10.3892/ol.2017.6776.

Zhang C, Ma M-H, Liang Y, Wu K-Z, Dai D-Q. 2019b. Novel long non-coding RNA LINC02532 promotes gastric cancer cell proliferation, migration, and invasion in vitro. *World journal of gastrointestinal oncology* 11:91–101. DOI: 10.4251/wjgo.v11.i2.91.

Zhang H, Qin D, Jiang Z, Zhang J. 2019c. SNHG9/miR-199a-5p/Wnt2 Axis Regulates Cell Growth and Aerobic Glycolysis in Glioblastoma. *Journal of neuropathology and experimental neurology* 78:939–948. DOI: 10.1093/jnen/nlz078.

Zhang Z, Wang S, Liu Y, Meng Z, Chen F. 2019d. Low lncRNA ZNF385D‑AS2 expression and its prognostic significance in liver cancer. *Oncology reports* 42:1110–1124. DOI: 10.3892/or.2019.7238.

Zhang F, Zeng L, Cai Q, Xu Z, Liu R, Zhong H, Mukiibi R, Deng L, Tang X, Xin H. 2020b. Comprehensive Analysis of a Long Noncoding RNA-Associated Competing Endogenous RNA Network in Wilms Tumor. *Cancer control: journal of the Moffitt Cancer Center* 27:1073274820936991. DOI: 10.1177/1073274820936991.

Zhang X, Zhang H, Fan C, Hildesjö C, Shen B, Sun X-F. 2022. Loss of CHGA Protein as a Potential Biomarker for Colon Cancer Diagnosis: A Study on Biomarker Discovery by Machine Learning and Confirmation by Immunohistochemistry in Colorectal Cancer Tissue Microarrays. *Cancers* 14. DOI: 10.3390/cancers14112664.

Zhang J, Zhang X, Piao C, Bi J, Zhang Z, Li Z, Kong C. 2019e. A long non-coding RNA signature to improve prognostic prediction in clear cell renal cell carcinoma. *Biomedicine & pharmacotherapy = Biomedecine & pharmacotherapie* 118:109079. DOI: 10.1016/j.biopha.2019.109079.

Zhang M, Zhang S, Wu L, Li D, Chen J. 2020c. Identification of potential prognostic biomarkers for tongue squamous cell carcinoma. *Research Square*. DOI: 10.21203/rs.3.rs-53695/v1.

Zheng B, Wang J, Fan K, Sun W, Wan W, Gao Z, Ni X, Zhang D, Ni X, Suo T, Liu H, Liu H, Shen S. 2021. lncRNA RP11-147L13.8 suppresses metastasis and chemo-resistance by modulating the phosphorylation of c-Jun protein in GBC. *Molecular therapy oncolytics* 23:124–137. DOI: 10.1016/j.omto.2021.08.016.

Zhong F, Zhu M, Gao K, Xu P, Yang H, Hu D, Cui D, Wang M, Xie X, Wei Y, Zhang H, Du H. 2018. Low expression of the long non-coding RNA NR_026827 in gastric cancer.

American journal of translational research 10:2706–2711.

Zhu L, Huang F, Wan T, Xu H, Zhao Q. 2018. Overexpression of long noncoding RNA LINC00882 is associated with poor prognosis in hepatocellular carcinoma. *OncoTargets and therapy* 11:5209–5217. DOI: 10.2147/OTT.S170825.

Zhu R, Li X, Cai Z, Liang S, Yuan Y, Xu Y, Lai D, Zhao H, Yang W, Bian J, Liu L, Xu G. 2021. Risk Scores Based on Six Survival-Related RNAs in a Competing Endogenous Network Composed of Differentially Expressed RNAs Between Clear Cell Renal Cell Carcinoma Patients Carrying Wild-Type or Mutant Von Hippel-Lindau Serve Well to Predict Malignancy and Prognosis. *Frontiers in oncology* 11:726671. DOI: 10.3389/fonc.2021.726671.

Zuo S, Wang L, Wen Y, Dai G. 2018. Identification of a universal 6-lncRNA prognostic signature for three pathologic subtypes of renal cell carcinoma. *Journal of cellular biochemistry*. DOI: 10.1002/jcb.28012.
