# Supplementary material for: Ectopic expression of HIV-1 Tat modifies gene expression in cultured B cells: implications for the development of B-cell lymphomas in HIV-1-infected patients
Source: PeerJ. 2022 Oct 18;10:e13986. doi: 10.7717/peerj.13986 (PMC9586123; doi:10.7717/peerj.13986)
Supplement: Supplemental Information 6 [file peerj-10-13986-s006.pdf]

**Table S5**

**Differential expression of MALAT1 and NEAT1 lncRNAs in B-cell-derived neoplasms (Lnc2cancer 3.0 database, http://bio-bigdata.hrbmu.edu.cn/lnc2cancer/).**

| LncRNA | Cancer name | Expression pattern | Reference |
|---|---|---|---|
| MALAT1 | chronic lymphocytic leukemia | up-regulated | (Ahmadi et al., 2018) |
| | mantle cell lymphoma | up-regulated | (Wang et al., 2016) |
| | myeloma | up-regulated | (Li et al., 2014; Cho et al., 2014; Ronchetti et al., 2016; Gao et al., 2017; Liu et al., 2017, 2020; Handa et al., 2017; Gu, Xiao & Yang, 2017; Hu et al., 2018; Sun et al., 2019) |
| | | down-regulated | (Isin et al., 2014) |
| | diffuse large B-cell lymphoma | up-regulated | (Li et al., 2017) |
| NEAT1 | chronic lymphocytic leukemia | up-regulated | (Blume et al., 2015; Ronchetti et al., 2020) |
| | multiple myeloma | up-regulated | (Wu & Wang, 2018; Taiana et al., 2020) |
| | diffuse large B-cell lymphoma | up-regulated | (Qian et al., 2020) |
| | Burkitt's lymphoma | up-regulated | (Blume et al., 2015) |

**References**

**Ahmadi A, Kaviani S, Yaghmaie M, Pashaiefar H, Ahmadvand M, Jalili M, Alimoghaddam K, Eslamijouybari M, Ghavamzadeh A. 2018.** Altered expression of MALAT1 lncRNA in chronic lymphocytic leukemia patients, correlation with cytogenetic findings. *Blood research* **53:**320–324. DOI: 10.5045/br.2018.53.4.320.

**Blume CJ, Hotz-Wagenblatt A, Hüllein J, Sellner L, Jethwa A, Stolz T, Slabicki M, Lee K, Sharathchandra A, Benner A, Dietrich S, Oakes CC, Dreger P, te Raa D, Kater AP, Jauch A, Merkel O, Oren M, Hielscher T, Zenz T. 2015.** p53-dependent non-coding RNA networks in chronic lymphocytic leukemia. *Leukemia* **29:**2015–2023. DOI: 10.1038/leu.2015.119.

**Cho S-F, Chang YC, Chang C-S, Lin S-F, Liu Y-C, Hsiao H-H, Chang J-G, Liu T-C. 2014.** MALAT1 long non-coding RNA is overexpressed in multiple myeloma and may serve as a marker to predict disease progression. *BMC cancer* **14:**809. DOI: 10.1186/1471-2407-14-809.

**Gao D, Lv A-E, Li H-P, Han D-H, Zhang Y-P. 2017.** LncRNA MALAT-1 Elevates HMGB1 to Promote Autophagy Resulting in Inhibition of Tumor Cell Apoptosis in Multiple Myeloma. *Journal of cellular biochemistry* **118:**3341–3348. DOI: 10.1002/jcb.25987.

**Gu Y, Xiao X, Yang S. 2017.** LncRNA MALAT1 acts as an oncogene in multiple myeloma through sponging miR-509-5p to modulate FOXP1 expression. *Oncotarget* **8:**101984–101993. DOI: 10.18632/oncotarget.21957.

**Handa H, Kuroda Y, Kimura K, Masuda Y, Hattori H, Alkebsi L, Matsumoto M, Kasamatsu T, Kobayashi N, Tahara K-I, Takizawa M, Koiso H, Ishizaki T, Shimizu H, Yokohama A, Tsukamoto N, Saito T, Murakami H. 2017.** Long non-coding RNA MALAT1 is an inducible stress response gene associated with extramedullary spread and poor prognosis of multiple myeloma. *British journal of haematology* **179:**449–460. DOI: 10.1111/bjh.14882.

**Hu Y, Lin J, Fang H, Fang J, Li C, Chen W, Liu S, Ondrejka S, Gong Z, Reu F, Maciejewski J, Yi Q, Zhao J-J. 2018.** Targeting the MALAT1/PARP1/LIG3 complex induces DNA damage and apoptosis in multiple myeloma. *Leukemia* **32:**2250–2262. DOI: 10.1038/s41375-018-0104-2.

**Isin M, Ozgur E, Cetin G, Erten N, Aktan M, Gezer U, Dalay N. 2014.** Investigation of circulating lncRNAs in B-cell neoplasms. *Clinica chimica acta; international journal of clinical chemistry* **431:**255–259. DOI: 10.1016/j.cca.2014.02.010.

**Li L-J, Chai Y, Guo X-J, Chu S-L, Zhang L-S. 2017.** The effects of the long non-coding RNA MALAT-1 regulated autophagy-related signaling pathway on chemotherapy resistance in diffuse large B-cell lymphoma. *Biomedicine & pharmacotherapy = Biomedecine & pharmacotherapie* **89:**939–948. DOI: 10.1016/j.biopha.2017.02.011.

**Li B, Chen P, Qu J, Shi L, Zhuang W, Fu J, Li J, Zhang X, Sun Y, Zhuang W. 2014.** Activation of LTBP3 gene by a long noncoding RNA (lncRNA) MALAT1 transcript in mesenchymal stem cells from multiple myeloma. *The Journal of biological chemistry* **289:**29365–29375. DOI: 10.1074/jbc.M114.572693.

**Liu N, Feng S, Li H, Chen X, Bai S, Liu Y. 2020.** Long non-coding RNA MALAT1 facilitates the tumorigenesis, invasion and glycolysis of multiple myeloma via miR-1271-5p/SOX13 axis. *Journal of cancer research and clinical oncology* **146:**367–379. DOI: 10.1007/s00432-020-03127-8.

**Liu H, Wang H, Wu B, Yao K, Liao A, Miao M, Li Y, Yang W. 2017.** Down-regulation of long non-coding RNA MALAT1 by RNA interference inhibits proliferation and induces apoptosis in multiple myeloma. *Clinical and experimental pharmacology & physiology* **44:**1032–1041. DOI: 10.1111/1440-1681.12804.

**Qian C-S, Li L-J, Huang H-W, Yang H-F, Wu D-P. 2020.** MYC-regulated lncRNA NEAT1 promotes B cell proliferation and lymphomagenesis via the miR-34b-5p-GLI1 pathway in diffuse large B-cell lymphoma. *Cancer cell international* **20:**87. DOI: 10.1186/s12935-020-1158-6.

**Ronchetti D, Agnelli L, Taiana E, Galletti S, Manzoni M, Todoerti K, Musto P, Strozzi F, Neri A. 2016.** Distinct lncRNA transcriptional fingerprints characterize progressive stages of multiple myeloma. *Oncotarget* **7:**14814–14830. DOI: 10.18632/oncotarget.7442.

**Ronchetti D, Favasuli V, Monti P, Cutrona G, Fabris S, Silvestris I, Agnelli L, Colombo M, Menichini P, Matis S, Gentile M, Nurtdinov R, Guigó R, Baldini L, Fronza G, Ferrarini M, Morabito F, Neri A, Taiana E. 2020.** NEAT1 Long Isoform Is Highly Expressed in Chronic Lymphocytic Leukemia Irrespectively of Cytogenetic Groups or Clinical Outcome. *Non-coding RNA* **6:**11. DOI: 10.3390/ncrna6010011.

**Sun Y, Jiang T, Jia Y, Zou J, Wang X, Gu W. 2019.** LncRNA MALAT1/miR-181a-5p affects the proliferation and adhesion of myeloma cells via regulation of Hippo-YAP signaling pathway. *Cell cycle* **18:**2509–2523. DOI: 10.1080/15384101.2019.1652034.

**Taiana E, Favasuli V, Ronchetti D, Todoerti K, Pelizzoni F, Manzoni M, Barbieri M, Fabris S, Silvestris I, Gallo Cantafio ME, Platonova N, Zuccalà V, Maltese L, Soncini D, Ruberti S, Cea M, Chiaramonte R, Amodio N, Tassone P, Agnelli L, Neri A. 2020.** Long non-coding RNA NEAT1 targeting impairs the DNA repair machinery and triggers anti-tumor activity in multiple myeloma. *Leukemia* **34:**234–244. DOI: 10.1038/s41375-019-0542-5.

**Wang X, Sehgal L, Jain N, Khashab T, Mathur R, Samaniego F. 2016.** LncRNA MALAT1 promotes development of mantle cell lymphoma by associating with EZH2. *Journal of translational medicine* **14:**346. DOI: 10.1186/s12967-016-1100-9.

**Wu Y, Wang H. 2018.** LncRNA NEAT1 promotes dexamethasone resistance in multiple myeloma by targeting miR-193a/MCL1 pathway. *Journal of biochemical and molecular toxicology* **32.** DOI: 10.1002/jbt.22008.
